# Metabolomic Profiling of Statin Use and Genetic Inhibition of HMG-CoA Reductase

**DOI:** 10.1016/j.jacc.2015.12.060

**Published:** 2016-03-15

**Authors:** Peter Würtz, Qin Wang, Pasi Soininen, Antti J. Kangas, Ghazaleh Fatemifar, Tuulia Tynkkynen, Mika Tiainen, Markus Perola, Therese Tillin, Alun D. Hughes, Pekka Mäntyselkä, Mika Kähönen, Terho Lehtimäki, Naveed Sattar, Aroon D. Hingorani, Juan-Pablo Casas, Veikko Salomaa, Mika Kivimäki, Marjo-Riitta Järvelin, George Davey Smith, Mauno Vanhala, Debbie A. Lawlor, Olli T. Raitakari, Nish Chaturvedi, Johannes Kettunen, Mika Ala-Korpela

**Affiliations:** a Computational Medicine, Institute of Health Sciences, University of Oulu and Biocenter Oulu, Oulu, Finland; b NMR Metabolomics Laboratory, School of Pharmacy, University of Eastern Finland, Kuopio, Finland; c Faculty of Epidemiology and Public Health, London School of Hygiene and Tropical Medicine, London, United Kingdom; d National Institute for Health and Welfare, Helsinki, Finland; e Institute for Molecular Medicine Finland, University of Helsinki, Helsinki, Finland; f University of Tartu, Estonian Genome Center, Tartu, Estonia; g Institute of Cardiovascular Science, University College London, London, United Kingdom; h Primary Health Care, School of Medicine, University of Eastern Finland and Kuopio University Hospital, Kuopio, Finland; i Department of Clinical Physiology, University of Tampere and Tampere University Hospital, Tampere, Finland; j Department of Clinical Chemistry, Fimlab Laboratories, and School of Medicine, University of Tampere, Tampere, Finland; k Institute of Cardiovascular and Medical Sciences, University of Glasgow, Glasgow, United Kingdom; l Department of Epidemiology and Public Health, University College London, London, United Kingdom; m Clinicum, Faculty of Medicine, University of Helsinki, Helsinki, Finland; n Institute of Health Sciences and Biocenter Oulu, University of Oulu, Oulu, Helsinki, Finland; o Department of Epidemiology and Biostatistics, MRC-PHE Centre for Environment and Health, Imperial College London, London, United Kingdom; p Oulu University Hospital, Oulu, Finland; q Medical Research Council Integrative Epidemiology Unit at the University of Bristol, Bristol, United Kingdom; r School of Social and Community Medicine, University of Bristol, Bristol, United Kingdom; s Primary Health Care, Central Finland Central Hospital, Jyväskylä, Finland; t Research Centre of Applied and Preventive Cardiovascular Medicine, University of Turku, Turku, Finland; u Department of Clinical Physiology and Nuclear Medicine, Turku University Hospital, Turku, Finland; v Computational Medicine, School of Social and Community Medicine, University of Bristol, Bristol, United Kingdom

**Keywords:** cholesterol lowering, drug development, lipoproteins, Mendelian randomization, metabolomics, CVD, cardiovascular disease, HDL, high-density lipoprotein, HMGCR, HMG-CoA reductase, IDL, intermediate-density lipoprotein, LDL-C, low-density lipoprotein cholesterol, NMR, nuclear magnetic resonance, VLDL, very-low-density lipoprotein

## Abstract

**Background:**

Statins are first-line therapy for cardiovascular disease prevention, but their systemic effects across lipoprotein subclasses, fatty acids, and circulating metabolites remain incompletely characterized.

**Objectives:**

This study sought to determine the molecular effects of statin therapy on multiple metabolic pathways.

**Methods:**

Metabolic profiles based on serum nuclear magnetic resonance metabolomics were quantified at 2 time points in 4 population-based cohorts from the United Kingdom and Finland (N = 5,590; 2.5 to 23.0 years of follow-up). Concentration changes in 80 lipid and metabolite measures during follow-up were compared between 716 individuals who started statin therapy and 4,874 persistent nonusers. To further understand the pharmacological effects of statins, we used Mendelian randomization to assess associations of a genetic variant known to mimic inhibition of HMG-CoA reductase (the intended drug target) with the same lipids and metabolites for 27,914 individuals from 8 population-based cohorts.

**Results:**

Starting statin therapy was associated with numerous lipoprotein and fatty acid changes, including substantial lowering of remnant cholesterol (80% relative to low-density lipoprotein cholesterol [LDL-C]), but only modest lowering of triglycerides (25% relative to LDL-C). Among fatty acids, omega-6 levels decreased the most (68% relative to LDL-C); other fatty acids were only modestly affected. No robust changes were observed for circulating amino acids, ketones, or glycolysis-related metabolites. The intricate metabolic changes associated with statin use closely matched the association pattern with *rs12916* in the *HMGCR* gene (*R*^2^ = 0.94, slope 1.00 ± 0.03).

**Conclusions:**

Statin use leads to extensive lipid changes beyond LDL-C and appears efficacious for lowering remnant cholesterol. Metabolomic profiling, however, suggested minimal effects on amino acids. The results exemplify how detailed metabolic characterization of genetic proxies for drug targets can inform indications, pleiotropic effects, and pharmacological mechanisms.

HMG-CoA reductase (HMGCR) inhibitors, commonly known as statins, reduce low-density lipoprotein cholesterol (LDL-C) levels leading to proportionate reduction in cardiovascular risk (1). Statins have become first-line therapy for managing dyslipidemia and cardiovascular disease (CVD) risk, making them the most widely prescribed drug class worldwide. Nearly 30% of Americans 45 years of age and older were receiving statins from 2007 to 2010 (2), and many more are eligible for treatment under the 2013 American College of Cardiology/American Heart Association cardiovascular prevention guidelines 3, 4.

Despite widespread use of statin therapy, their effects on many lipids and other metabolic biomarkers of cardiovascular risk, such as circulating fatty acids and amino acids 5, 6, have not been assessed in large studies. Statins have been proposed to possess various pleiotropic properties such as reducing inflammation and improving endothelial function 7, 8, yet it remains unclear whether such effects would manifest in the systemic metabolic profile. Although the vascular event rate reduction follows a linear relationship with LDL-C lowering (9), the cardioprotective abilities of statins may also partly be attributed to other lipids 10, 11, 12. Of particular importance are the effects on triglycerides and remnant cholesterol, because these measures have been causally linked to the development of coronary heart disease 13, 14, 15. Direct assaying of remnant cholesterol, that is, the cholesterol in very-low-density lipoprotein (VLDL) and intermediate-density lipoprotein (IDL) particles, has recently become feasible as part of the lipoprotein subclass profiling provided by nuclear magnetic resonance (NMR) metabolomics (16). This high-throughput profiling simultaneously quantifies numerous other biomarkers, which, in concert, provide a fine-grained snapshot of systemic metabolism 5, 17.

We aimed to determine comprehensive metabolic effects of statin therapy by conducting metabolomic profiling at 2 time points in 4 population-based cohorts. To verify that the observed lipoprotein, fatty acid, and metabolite changes are due to the effects of statins, the results were corroborated via Mendelian randomization by using a genetic variant in the *HMGCR* gene as a proxy for the pharmacological action of statins 18, 19, 20. Specifically, we examined the metabolic effects of genetic variation in *HMGCR* (19)—mimicking a very small dose of statin allocated to rs12196-T carriers, and unaffected by confounding due to the random assorting of alleles at conception (18)—and compared the genetic association pattern to the metabolic changes observed longitudinally.

## Methods

### Study populations

All study participants provided written informed consent, and study protocols were approved by the local ethics committees. The metabolic changes associated with starting statin therapy were examined in 4 U.K.-based and Finnish longitudinal cohorts with NMR-based metabolomics data from overnight fasting samples at baseline and a follow-up visit: the SABRE study (Southall and Brent Revisited; 20 to 23 years of follow-up during 1988 to 2011; N = 908) 5, 21, the Pieksämäki Cohort (6-year follow-up, 1997 to 2003; N = 608) (22), the YFS (Cardiovascular Risk in Young Finns Study; 4-year follow-up, 2007 to 2011; N = 1,562) 22, 23, and the mothers cohort of the ALSPAC study (Avon Longitudinal Study of Parents and Children; 2.5-years of follow-up, 2009 to 2011; N = 2,452) (24). Details of the cohorts are described in the Online Appendix. Information on statin use was obtained from questionnaires. Data on specific statin type and dose were generally not available. Individuals on non-statin lipid-lowering monotherapy (12 subjects) and pregnant women were omitted from the analyses. Altogether 5,590 individuals with metabolomic profile measured at both time points and free of statin medication at baseline were included in the longitudinal analyses.

For Mendelian randomization, we analyzed *rs12916* in the *HMGCR* gene, a genetic variant known to affect hepatic HMGCR expression and circulating LDL-C 13, 19, in 8 population-based cohorts from the United Kingdom and Finland with metabolomics data from the same NMR platform: ALSPAC children (n = 2,456) (25) and mothers (n = 3,137) (24), NFBC (Northern Finland Birth Cohort) 1986 (N = 4,145) (26) and NFBC 1966 (N = 4,920) (27), YFS (N = 1,905) (23), the FINRISK 1997 study (N = 4,403) (5), the British Women’s Heart and Health Study (N = 3,030) (5), and the Whitehall II study (N = 3,918) (28) (detailed in the Online Appendix). Pregnant women and individuals on lipid-lowering treatment were excluded from analyses. Altogether, 27,914 individuals with metabolomics data at a single time point and *rs12916* genotype information were available for the Mendelian randomization analyses. We further confirmed the metabolic association pattern with *rs17238484* in *HMGCR,* which is in low linkage disequilibrium (*R*^2^ = 0.37) with *rs12916* but affects LDL-C to a similar extent (19).

### Lipoprotein, fatty acid, and metabolite quantification by metabolomics

A high-throughput NMR metabolomics platform 17, 29 was used to quantify 80 lipid and low-molecular-weight metabolite measures from serum or plasma samples in 4 longitudinal cohorts at 2 time points and 8 population-based cohorts with *HMGCR* genotype information. This platform provided simultaneous quantification of routine lipids, particle concentrations of 14 lipoprotein subclasses; lipid concentrations in major subfractions; and further abundant fatty acids, amino acids, ketone bodies, and various glycolysis- and gluconeogenesis-related metabolites in absolute concentration units (Online Table 1) 5, 6, 17, 22, 30, 31. The NMR metabolomics platform has been extensively used in epidemiological and genetic studies 5, 17, 22, 30, 31, and the experimentation has been described elsewhere 17, 29. NMR spectral data from 3 molecular windows with annotated metabolites are illustrated (Online Figure 1) for a representative individual before and after starting statin therapy; however, all statistical analyses of statin effects were conducted on the quantitative biomarker measures, and no analysis directly on the spectral data was performed.

### Statistical analysis

The effects of statin therapy were examined by comparing metabolic changes for those who started statins during follow-up to the changes observed for persistent nonusers. The mean difference in metabolite concentration change between the statin-starter group and the nonuser group was assessed by linear regression models adjusted for age and sex. Analyses were conducted separately for each cohort and meta-analyzed using inverse variance–weighted fixed effects. To enable comparison of association magnitudes across measures with different units and distinct relation to cardiovascular risk, all lipid and metabolite concentrations were scaled to baseline SD units. The differences in concentration change between statin starters and nonusers are therefore reported in SD units; the corresponding absolute concentration changes are listed in Online Table 1. To facilitate comparison with the genetic analyses, longitudinal association magnitudes are also shown scaled to the lowering effect on LDL-C. The metabolic changes in percentage relative to baseline concentrations were examined as secondary analyses. Statistical significance was denoted at p < 0.0006 to account for the testing of 80 metabolic measures.

For genetic analyses, lipid and metabolite concentrations were first adjusted for age, sex, and the first 4 genomic principal components, and then inverse normal transformed to enhance statistical power (30). Subsequently, *rs12916* in *HMGCR* was tested for association with each metabolic measure as outcome using linear regression. Results were assessed separately for each cohort and meta-analyzed using inverse variance–weighted fixed effects. Effect sizes are presented in SD units difference in concentration per *rs12916-T* allele. For comparison with longitudinal metabolic changes, the results are also shown scaled to the effect of *rs12916-T* on LDL-C. The overall match between genetic and longitudinal associations was summarized by the linear fit of the correspondence between metabolic association patterns, with both scaled relative to the effect on LDL-C (22). Statistical analyses were conducted using R version 3.2 (R Foundation for Statistical Computing, Vienna, Austria).

## Results

Among 5,590 participants from 4 population-based cohorts with metabolomic profiling at 2 time points (Table 1), 716 subjects started statin therapy during follow-up. The metabolic effects of statin use were quantified by comparing lipid and metabolite changes between the statin starters and the persistent nonusers during follow-up. To obtain an unconfounded assessment of the on-target effects of statins, we further examined lipid and metabolite associations with *rs12916* in *HMGCR* for 27,914 individuals from 8 population-based cohorts (Online Table 2).

**Table 1:** Baseline Characteristics

	Southall and Brent REvisited (SABRE) Study		Pieksämäki Cohort Study		Cardiovascular Risk in Young Finns Study		Avon Longitudinal Study of Parents and Children (Mothers)	
	Nonusers (n = 372)	Starters (n = 536)	Nonusers (n = 562)	Starters (n = 106)	Nonusers (n = 1,519)	Starters (n = 43)	Nonusers (n = 2,421)	Starters (n = 31)
Follow-up time, yrs	20–23		6–7		4–5		2–3	
Male	84	89	41	43	44	63	0	0
Age, yrs	48.5 ± 6.1	50.3 ± 6.3	45.4 ± 6.2	48.5 ± 5.4	37.9 ± 5.0	40.7 ± 4.0	48.2 ± 4.3	50.7 ± 4.8
BMI, kg/m^2^	25.1 ± 3.1	26.3 ± 3.6	26.1 ± 4.1	27.9 ± 6.2	25.8 ± 4.6	28.9 ± 5.9	26.0 ± 4.9	28.2 ± 4.5
Systolic blood pressure, mm Hg	117 ± 15	124 ± 16	134 ± 18	138 ± 18	120 ± 14	129 ± 13	118 ± 12	128 ± 17
Plasma glucose, mmol/l	5.3 (4.9–5.7)	5.5 (5.1–5.9)	5.6 (5.3–6.0)	5.8 (5.5–6.3)	5.2 (4.9–5.6)	5.4 (5.2–5.8)	5.1 (4.9–5.4)	5.3 (5.1–5.8)
HDL cholesterol, mmol/l	1.3 ± 0.3	1.2 ± 0.3	1.4 ± 0.3	1.4 ± 0.3	1.3 ± 0.3	1.2 ± 0.4	1.5 ± 0.4	1.3 ± 0.4
Friedewald LDL cholesterol, mmol/l	3.6 ± 0.9	4.1 ± 1.0	3.5 ± 0.8	4.3 ± 0.9	3.1 ± 0.8	4.1 ± 0.8	3.0 ± 0.8	4.1 ± 1.3
Total cholesterol, mmol/l	5.6 ± 1.0	6.3 ± 1.1	5.5 ± 0.9	6.4 ± 0.9	5.0 ± 0.9	6.0 ± 0.9	4.9 ± 0.8	6.1 ± 1.4
Triglycerides, mmol/l	1.2 (0.9–1.7)	1.7 (1.2–2.5)	1.1 (0.8–1.6)	1.4 (1.1–2.0)	1.1 (0.8–1.6)	1.6 (1.1–2.3)	0.8 (0.7–1.1)	1.3 (1.0–2.0)

Values are %, mean ± SD, or median (interquartile range), unless otherwise indicated. Characteristics of the 8 population-based cohorts used for genetic analyses are shown in Online Table 2.

BMI = body mass index; HDL = high-density lipoprotein; LDL = low-density lipoprotein.

### Statin effects

**Figure 1:**
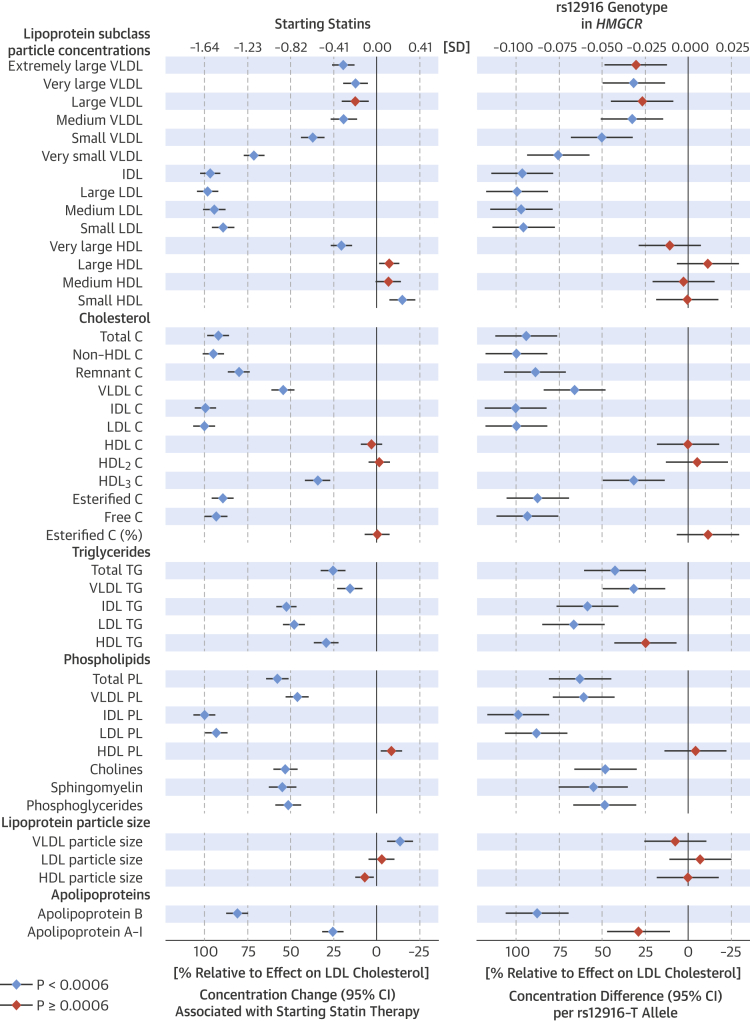
Lipoprotein and Lipid Associations **(Left)** Lipoprotein and lipid changes associated with starting statin therapy (n = 716) compared with the corresponding changes for persistent nonusers (n = 4,874) during follow-up. Associations were adjusted for age and sex, and meta-analyzed for 4 longitudinal cohorts. **(Right)** Lipoprotein and lipid associations with *rs12916* in *HMGCR* adjusted for age, sex, and population stratification meta-analyzed for 8 cohorts (N = 27,914). **Error bars** = 95% confidence intervals (CI). Results are shown in SD-scaled concentration units **(top axis)** and relative to the lowering effect on low-density lipoprotein (LDL) cholesterol **(bottom axis)**. Changes in absolute concentration units are listed in Online Table 1, and in percentage relative to baseline levels in Online Figure 2. C = cholesterol; CI = confidence interval; HDL = high-density lipoprotein; IDL = intermediate-density lipoprotein; LDL = low-density lipoprotein; PL = phospholipids; VLDL = very-low-density lipoprotein; TG = triglycerides.

The changes of 44 lipoprotein measures associated with starting statin therapy and the corresponding differences per *rs12916-T* allele are shown (Figure 1). To facilitate comparison between longitudinal and genetic effects, association magnitudes are shown scaled to the lowering effect on LDL-C (1.65 SD for starting statins; 0.096 SD per *rs12916-T* allele). The changes associated with starting statins followed a strikingly similar pattern as the associations with *HMGCR* genotype across all lipoprotein measures. Starting statins was associated with minor lowering of large- and medium-sized VLDL particle concentrations (11% to 20% relative to the LDL-C-lowering effect), whereas substantial lowering of the smallest VLDL particles (71% relative to LDL-C) was observed. The lowering of particle concentrations was similar across LDL subclasses and IDL (94% to 100%). Starting statins was associated with a modest lowering of very large high-density lipoprotein (HDL) particle concentrations, whereas the concentration of small HDL particles was modestly increased. Large- and medium-sized HDL particle concentrations were essentially unaffected. Total cholesterol, non–HDL-C, and IDL-C were lowered to a similar degree as LDL-C (92% to 100%); lowering of VLDL-C was less (54%). Remnant cholesterol was lowered to a similar extent as apolipoprotein B (80%). By contrast, statin use was associated with modest lowering of VLDL and total triglycerides (15% and 25%, respectively). More pronounced lowering was observed for IDL and LDL triglycerides (52% and 49%). Starting statin therapy was only weakly associated with lipoprotein particle size. All lipid and metabolite changes associated with starting statin therapy are listed in absolute concentration units (e.g., mmol/l) in Online Table 1. The metabolic changes in percentage relative to baseline concentrations are shown in Online Figure 2.

**Figure 2:**
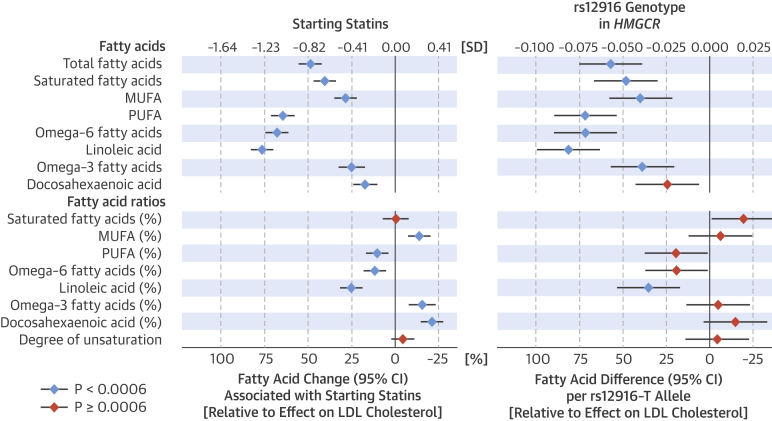
Fatty Acid Associations **(Left)** Fatty acid changes associated with starting statin therapy compared to the corresponding changes for persistent nonusers during follow-up and **(right)** fatty acid associations with *rs12916* in *HMGCR*. MUFA = monounsaturated fatty acids; PUFA = polyunsaturated fatty acids; other abbreviations as in Figure 1.

The changes in circulating fatty acid levels associated with starting statin therapy and the genetic proxy of HMGCR inhibition displayed a matching pattern (Figure 2), with similar magnitudes relative to the extent of LDL-C lowering. The absolute concentrations of all assayed fatty acids were lowered in the statin group compared to nonusers (18% to 77% relative to the SD-scaled lowering of LDL-C), with prominent differences between different fatty acid types. Absolute levels of saturated and monounsaturated fatty acids were lowered to a lesser extent than total fatty acids (49% relative to LDL-C), but only minor changes were observed in their ratios to total fatty acids. Omega-6 fatty acids, including linoleic acid, displayed the most pronounced lowering associated with statin use, and the ratio of these measures to total fatty acids was also decreased. By contrast, statin use was only weakly associated with lowering of omega-3 fatty acids, including docosahexaenoic acid, which resulted in a modest increase in their ratio to total fatty acids.

**Figure 3:**
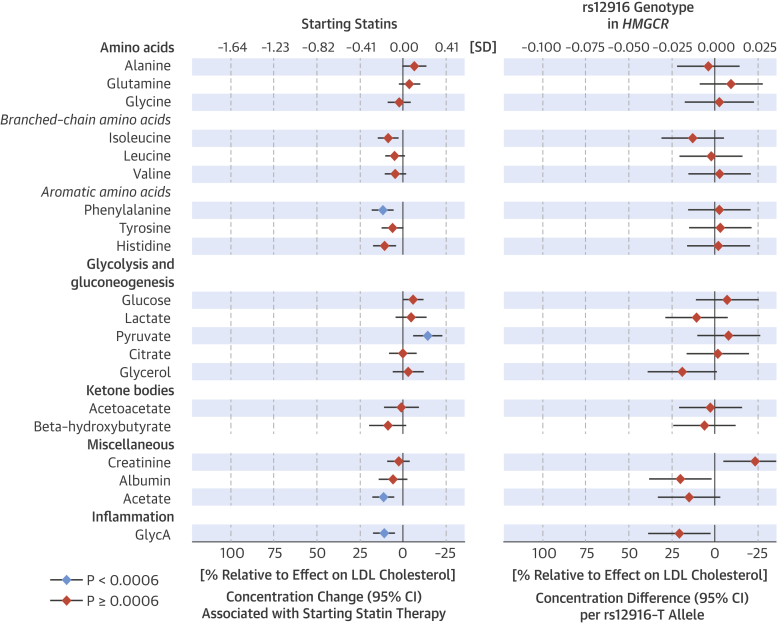
Metabolite Associations **(Left)** Metabolite changes associated with starting statin therapy compared to the corresponding changes for persistent nonusers during follow-up and **(right)** metabolite differences associated with *rs12916* in *HMGCR*. GlycA = glycoprotein acetyls; other abbreviations as in Figure 1.

To assess potential nonlipid effects of statin use, we examined the changes in circulating amino acids, glycolysis and gluconeogenesis substrates and products, ketone bodies, and other metabolites quantified by the high-throughput metabolomics platform (Figure 3). Starting statins was only weakly or negligibly associated with these metabolites (maximum 12% lowering to 14% increase, relative to the effect on LDL-C). The corresponding associations of *rs12916* in *HMGCR* with these metabolites did not coherently match the weak observational associations. The only deviations from this pattern were a small decrease in glycoprotein acetyl (GlycA) (a marker of low-grade inflammation) 32, 33, and acetate concentrations, which decreased both observationally and genetically.

### Genetic and observational consistency

**Figure 4:**
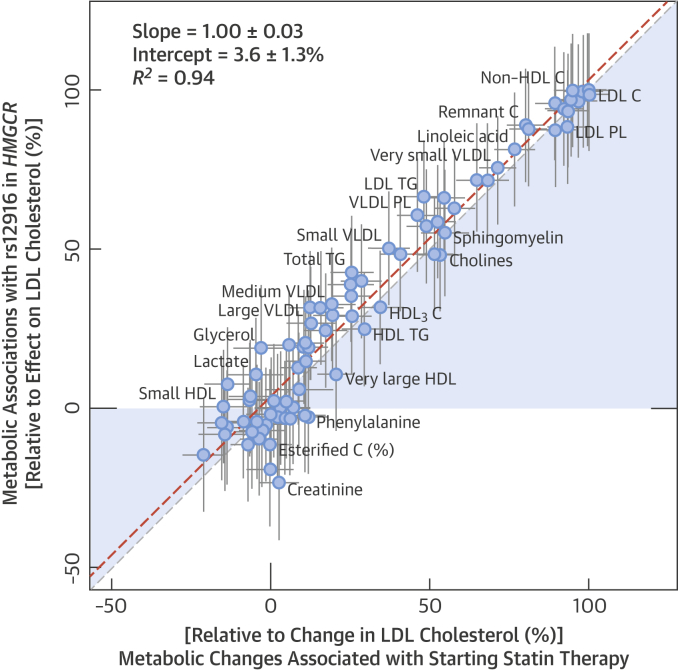
Correlation Between Metabolic Changes Both longitudinal and genetic association magnitudes are scaled relative to the lowering effect on LDL cholesterol. **Dashed line** = linear fit between longitudinal and genetic associations. *R*^2^ = goodness of fit. Abbreviations as in Figure 1.

The overall match between the metabolic changes associated with starting statins and the corresponding associations with the *HMGCR* variant is illustrated in Figure 4. The longitudinal and genetic association magnitudes fell closely on a straight line (*R*^2^ = 0.94); the slope of the fit was 1.00 ± 0.03 when both genetic and longitudinal associations were scaled to the lowering effect on LDL-C. The Central Illustration further depicts use of a genetic variant to validate the causal molecular effects of HMGCR inhibition across multiple metabolic pathways.

**Central Illustration:**
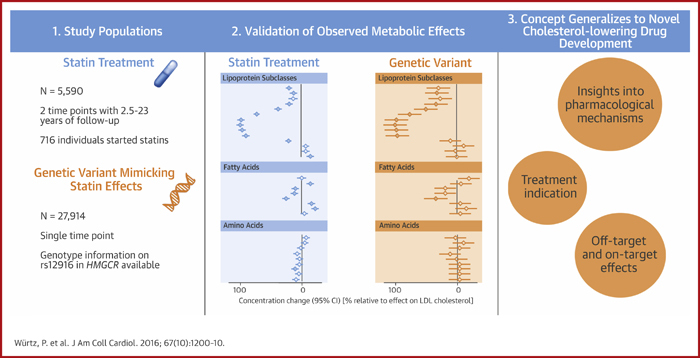
Metabolomic Profiling of Statin Therapy: Longitudinal Cohorts and an *HMGCR* Gene Variant Mimicking the Statin Effect As assessed from longitudinal cohorts and an *HMGCR* gene variant mimicking the effect of statins, widespread lipid-lowering occurs beyond low-density lipoprotein cholesterol (LDL-C), but there is minimal evidence for similar effects on non-lipid metabolites. **Blue diamonds** represent longitudinal metabolic changes associated with starting statins. **Orange diamonds** represent metabolic associations with *rs12916* in *HMGCR*, denoting the causal effects of HMGCR inhibition. Association magnitudes are relative to the lowering effect on LDL-C. CI = confidence interval.

The pattern of metabolic changes associated with starting statins was similar if calculated in percentage changes relative to baseline levels (Online Figure 2). Despite substantial differences in follow-up time and demographics across the 4 longitudinal cohorts, the metabolic changes were consistent between the studies (Online Figure 3). The results were essentially unaltered when further adjusted for change in body mass index during follow-up (Online Figure 4). The results were also similar if adjusting for additional cardiovascular risk factors, including baseline LDL-C (Online Figure 4). The genetic association pattern was coherent across the 8 cohorts analyzed (Online Figure 3). All lipid and metabolite associations were similar if using rs17238484 instead of *rs12916* as a proxy for HMGCR inhibition (Online Figure 5).

## Discussion

Metabolomic profiling of statin use in longitudinal cohorts uncovered an intricate association pattern of circulating lipoprotein, fatty acid, and metabolite changes, which adds to our understanding of the LDL-C–independent effects of statins. Statin use was associated with pronounced lowering of numerous lipids and fatty acids consistent with the cardioprotective effects. By contrast, statin use did not markedly affect the circulating levels of recently identified biomarkers for cardiometabolic risk such as amino acids, glycolysis- and glycogenesis-related metabolites, or ketone bodies 5, 6, 21, 31. The genetic proxy for HMGCR inhibition gave rise to a strikingly similar association pattern, providing unconfounded evidence that the observed metabolic changes arise as a consequence of the mechanism-based effect of statins (Central Illustration). These insights into an extensively studied therapeutic agent illustrate how metabolomics, combined with genetic proxies mimicking pharmacological action, can elucidate the molecular effects of known targets, clarify treatment indication, and potentially be used to inform drug development 19, 20, 34.

Inhibition of HMGCR by statins leads to up-regulated expression of LDL receptors in the liver, which in turn increases the uptake of circulating LDL particles. Using lipoprotein subclass profiling, statin therapy also was shown to be associated with considerable lowering of IDL and very small VLDL particle concentrations, beyond the anticipated decrease in LDL particles. These remnant lipoprotein particles carry 20% to 30% of circulating cholesterol; they are small enough to enter the arterial intima and, therefore, possess the potential to cause atherosclerosis (14). Because total triglyceride concentration is highly correlated with the amount of IDL and small VLDL particles and their cholesterol levels, the triglyceride measure may partly reflect the cardiovascular risk mediated by the atherogenic remnant particles 14, 15. Indeed, accumulating genetic evidence suggests that triglyceride levels reflect causal processes related to coronary heart disease 13, 14, 15; however, the likely underpinning mechanism is the remnant cholesterol carried in the IDL and VLDL particles 14, 15, 35. Detailed lipoprotein profiling demonstrated that statins are effective in lowering remnant cholesterol, whereas triglycerides are only modestly decreased by statin therapy. These results suggest that statins are substantially more efficacious for lowering remnant cholesterol than would be projected based on their ability to lower triglycerides. If the cardiovascular risk reflected by triglycerides is due to remnant cholesterol rather than triglycerides per se 14, 35, then our results indicate cardioprotective benefits of statins beyond LDL-C lowering and suggest broader indications for statins in treating remnant hyperlipidemia.

The fatty acid composition of lipoprotein lipids vary greatly depending on the abundance of cholesteryl esters, triglycerides, and phospholipids (36). In accordance with linoleic acid being the primary constituent of cholesteryl esters—the dominant lipid in LDL particles—statin therapy led to the greatest lowering of this omega-6 fatty acid. Absolute levels of omega-3 fatty acids were only modestly decreased, in agreement with prior studies 12, 37. These results are consistent with omega-3 fatty acids being primarily bound to the phospholipids, which only account for some 30% of the lipids in LDL particles (36). Monounsaturated and saturated fatty acids were decreased to a broadly similar extent as total triglycerides and phospholipids, respectively, which is coherent with the main fatty acid compositions for these lipid classes (36). The changes in the relative fatty acid balance due to statin therapy were modest. Although lower levels of the ratio of omega-6 fatty acids to total fatty acids have been associated with higher cardiovascular risk 5, 38, evidence for a causal relation is lacking. The overall consistency between the genetic and longitudinal association patterns indicate that the various fatty acid modulations are on-target effects of HMGCR inhibition rather than due to cholesterol-independent properties of statins (37).

We also assessed whether statin therapy would be associated with biomarkers in various nonlipid pathways. GlycA, a measure of systemic inflammation and a biomarker for CVD and all-cause mortality 5, 6, 32, 33, 39, was modestly lowered, in accordance with the proposed anti-inflammatory properties of statins 7, 8. However, both longitudinal and genetic analyses provided no evidence for substantial effects of statins on amino acids, glycolysis and gluconeogenesis metabolites, and ketone bodies. Several metabolites in these pathways have recently been shown to be risk markers for CVD and type 2 diabetes 5, 6, 17, 21, 31. Although the potential causal roles of these biomarkers remain unclear, our results suggested that statin therapy would not be efficacious for lowering the cardiometabolic risk associated with these markers.

### Study limitations

The observational assessment of the effects of statins may be confounded, in particular by indication for treatment. However, the comparisons of metabolic changes over 2 time points reduced such confounding. Furthermore, the Mendelian randomization approach to proxy the metabolic effects of HMGCR inhibition is generally free of this limitation 18, 40. The *rs12916-T* allele in *HMGCR* has previously been rigorously associated with lower expression of HMGCR in the liver and lower circulating LDL-C levels 13, 19, supporting the validity of this common variant as a genetic instrument. Information on statin type and dosage was generally not available; however, results were coherent across the 4 longitudinal cohorts despite large differences in demographics and follow-up time. The genetic analyses were also consistent across 8 cohorts with a wide age span. The limited statistical power and the predominantly young study population preclude us from ruling out minor effects of statins on nonlipid biomarkers. Nonetheless, the results set upper limits for the effects of HMGCR inhibition on multiple circulating biomarkers not previously investigated.

## Conclusions

High-throughput metabolomic profiling in large cohorts with multiple time points and genetic information elucidated the pharmacological effects of statins on lipoprotein subclasses including their lipid constituents and fatty acid composition. These results suggest a more efficacious role of statins for lowering remnant cholesterol levels than would be expected based on the ability of statins to lower circulating triglycerides. The absence of robust associations of statin use with circulating amino acids, glycolysis and gluconeogenesis metabolites, and ketone bodies suggest minimal pleiotropic effects on these nonlipid biomarkers. As a corollary, statin therapy appears to have little or no efficacy on these novel markers of cardiometabolic risk. The exquisite match between the metabolic association patterns from observational and genetic analyses serves as a proof of concept, illustrating how the combination of metabolomics and genetic proxies for drug mechanisms can facilitate the assessment of pharmacological action and on-target effects for known therapies and novel drug targets.

Although Mendelian randomization of drug targets has been used previously 19, 20, 34, our study was the first to our knowledge to combine the concept with observational results across a wide range of cardiometabolic biomarkers. As extensive metabolomics and genetic data are increasingly becoming available in large biobanks, such comprehensive molecular profiling can augment drug development in both preclinical and clinical trial stages to elucidate molecular mechanisms, clarify pleiotropic effects, and inform treatment indication.

Perspectives **COMPETENCY IN MEDICAL KNOWLEDGE:** Longitudinal studies and Mendelian randomization suggest that statin therapy lowers blood levels of remnant cholesterol, but has modest effects on fatty acid ratios and minimal effects on circulating amino acids.

**TRANSLATIONAL OUTLOOK:** Metabolic profiling, combined with genetic proxies mimicking pharmacological action, may prove useful to elucidate the molecular effects of known and novel drugs.
