# Supplementary material for: Metabolomic Profiling of Statin Use and Genetic Inhibition of HMG-CoA Reductase
Source: J Am Coll Cardiol. 2016 Mar 15;67(10):1200–10. doi: 10.1016/j.jacc.2015.12.060 (PMC4783625; doi:10.1016/j.jacc.2015.12.060)
Supplement: Online Data [file mmc1.pdf]

SUPPLEMENTAL MATERIAL

Würtz, Wang, Soininen, et al. Metabolomic profiling of statin use and genetic inhibition of HMG-CoA reductase.

Supplementary Methods.

Supplementary Table 1. Mean absolute concentrations of lipoprotein, fatty acid and metabolite measures in the study population, and absolute concentration changes associated with starting statin therapy and rs12916 in *HMGCR.*

Supplementary Table 2. Clinical characteristics of the eight study populations used for the genetic analyses stratified by rs12916 genotype.

Supplementary Figure 1. Representative NMR spectra before and after statin use.

Supplementary Figure 2. Lipid and metabolite changes associated with starting statin therapy relative to baseline concentrations.

Supplementary Figure 3A-F. Lipid and metabolite changes associated with starting statin therapy and rs12196 in *HMGCR* separately for the individual cohorts.

Supplementary Figure 4. Lipid and metabolite changes associated with starting statin therapy when adjusted for additional cardiovascular risk factors.

Supplementary Figure 5. Comparison of lipid and metabolite associations for two common genetic variants in the *HMGCR* gene.

Supplementary Methods

Study populations

Longitudinal analyses: Lipid and metabolite associations with starting statin therapy

Southall and Brent Revisited (SABRE)

The SABRE study examined 4,857 individuals in a tri-ethnic community-based cohort from North and West London, UK (www.sabrestudy.org). Details of the cohort have been published.[1,2] Briefly, participants aged 40 to 69 at baseline (1988 through 1991) were selected randomly from 5-year age- and sex-stratified primary care physician lists. Participants included Europeans (48%), Indian Asians (47%) and a small fraction of African Caribbeans (5%). Participants attended a baseline clinic after an overnight fast. Fasting blood samples were drawn for Southall participants and stored at −80°C for biomarker measurements. Study participants were invited for a follow-up visit during 2008 through 2011. NMR metabolomics was measured for 3,298 baseline serum samples with available stored aliquots.[3] Among these, 919 attended the follow-up survey in 2008-2011, and had the serum metabolomic profiling done also at the follow-up.[4,5] None of these study participants used lipid-lowering therapy at the 1988-1991 baseline. Study questionnaires asked for the use of medication, including the type of medication; 98.3% out of the 536 individuals on lipid-lowering medication at follow-up were using statins. The seven individuals on ezetimibe and four individuals on fibrate mono-therapy were omitted, leaving 908 individuals for the present analyses. Information on *HMGCR* genotype was not available in this cohort. All participants gave written informed consent. The study protocols were approved by the University College London and St. Mary's Hospital research ethics committees.

The Pieksämäki cohort

The Pieksämäki cohort study consisted of individuals from the town of Pieksämäki, Eastern Finland, born in 1942, 1947, 1952, 1957 and 1962. There were 923 participants in the initial examination in 1997, and 690 of these attended a follow-up survey 6.5-years later (2003–2004).[4,5] In the present study, 668 individuals with NMR-based metabolomics measured from fasting serum samples at both time-points, and free of statin or other lipid-lowering medication (n=16 excluded) at baseline, were analyzed. Among these, 106 had initiated statin therapy during follow-up. No genotype information was available in this cohort. Participants gave written informed consent and the study protocol was approved by the Ethics Committee of Kuopio University Hospital, Finland.

The Cardiovascular Risk in Young Finns Study (YFS)

The Cardiovascular Risk in Young Finns Study was initiated to study associations of childhood risk factors to cardiovascular disease in adulthood (youngfinnsstudy.utu.fi).[6] The baseline study in 1980 included 3,596 children and adolescents aged 3–18. Metabolomics data in the longitudinal analyses of the present study are based on the 2007→2011 surveys with 2,160 and 2,040 participants having metabolomics measured from fasting serum samples collected in 2007 and 2011, respectively.[7,8] Among these, 1,744 participants had their metabolomic profile measured from both surveys. After exclusion of individuals on lipid-lowering medication at baseline (n=37) or missing treatment information (n=105) and pregnant women (n=39), the present study comprised 1,562 individuals with an NMR-based metabolomics measured at both time-points. These individuals were representative of the original cohort.[7] Study questionnaires addressed the type of lipid-lowering medication. At 4-year follow-up, 43 individuals had initiated statin therapy. A single individual reporting to use only non-statin lipid-lowering treatment was excluded, whereas two individuals reporting to initiate combination therapy were counted as statin starters.

In addition to longitudinal data, YFS also contributed to the Mendelian randomization analyses of *HMGCR*. Genotype information on the two genetic variants (rs12196 and rs17238484) in *HMGCR* was available for n=1,905 participants from the 2007 field study after exclusion of

individuals on lipid-lowering medication and pregnant women at this ~~timepoint~~. Genotype data was based on 670k Illumina HumanHap arrays.[8] Both common single nucleotide polymorphisms (SNPs) were imputed based on the 1000 Genome reference panel. All participants gave written informed consent. The study was approved by the ethics committees of each of the five participating medical university study sites in Finland.

The Avon Longitudinal Study of Parents and Children (ALSPAC) – Mothers Cohort
The Avon Longitudinal Study of Parents and Children was established to understand how genetic and environmental characteristics influence health and development in parents and children (bristol.ac.uk/alspac).[9] All pregnant women resident in a defined area in the South West of England, with an expected date of delivery between 1st April 1991 and 31st December 1992, were eligible and 13,761 women were recruited. These mothers have been followed over more than two decades.[9] NMR-based metabolomics was measured from plasma samples collected during two follow-up clinic assessments; the first occurring between 2009 and 2011 (17-19 years after the index pregnancies) and the second 2011-2012 (19-20 years after the index pregnancies). For the first assessment, about 10,000 women who remained engaged with the study were invited and 50% attended. For the second a 3,000 subsample of the first assessment who were anticipated to go through a menopausal status transition were invited and over 85% attended. At both assessments women attended after an overnight fast for those attending in the morning or minimum 6-hours fast for those attending in the afternoon.[9]

Metabolomics data were measured from stored EDTA plasma samples for 4,524 women in the first assessment and 2,749 women in the second assessment. Altogether 2,487 women had their metabolomic profile measured at both time-points. Thirty-five of these women were excluded because they were using statins at the first (baseline) assessment, resulting in 2,452 women with NMR-based metabolomics measured at both time-points included in the present analyses.

During the clinical assessments participants were asked to provide details of all medications that they were using, including bringing medicine containers and prescriptions. All medications were entered in a database with text names and information on dosage and frequency and the reason the woman gave for taking the medication. The medication data was encoded to indicate which women were taking statins at each time point. This was done by searching for all statin names that appeared in the 2014 electronic version of the British National Formulary. This was supplemented by searching for any reference to 'cholesterol', 'blood fats', 'heart disease', 'heart attack', 'angina' and 'heart' in text entries to identify common misspellings of drug names, which were then used to undertake additional searches. No other lipid lowering drugs were used in the cohort.

In addition to longitudinal data, ALSPAC mothers also contributed to the Mendelian randomization analyses of *HMGCR*. Genotype information and population stratification was obtained from Illumina Human660W-Quad BeadChips run at Centre National de Génotypage, Evry, France. Data on genotype and metabolomic profile from the first (2009-2011) follow-up assessment were used in the Mendelian randomization analyses with these data available on 3,137 women after exclusion of 53 on statin therapy. rs698912 was used as proxy for the validation variant rs17238484, linkage disequilibrium R^2=1. The study was approved by the ALSPAC Law and Ethics Committee and the UK National Health Service Research Ethics Committee and all participants provided written informed consent.

Genetic analyses: Lipid and metabolite associations with common variants in *HMGCR*

Northern Finland Birth Cohort of 1986 (NFBC 1986)
The Northern Finland Birth Cohorts of 1986 and 1966 were initiated to study factors affecting preterm birth and subsequent morbidity in the two northernmost provinces in Finland (www.oulu.fi/nfbc). For NFBC 1986, the number of deliveries in the birth cohort was 9,362, which was 99% of all the deliveries taking place in the area during the target period (July 1985-June

1986).[10,11] Data collection in 2001–2002 included clinical examination and serum sampling at age 15–16 for 6,621 adolescents; attendees in the 16-year field study (71%) were representative of the original cohort.[12] Metabolomics data from this timepoint were used for the genetic analyses in the present study. NMR metabolomics was measured for 5,602 serum samples, of which 95% were drawn after overnight fasting.[8] Among these, 4,145 individuals had information available on the rs12196 variant in *HMGCR* from the CardioMetabochip and genomic information on population stratification. The SNP rs3761742 was used as proxy for the validation variant rs17238484 (linkage disequilibrium R^2=1). Informed written consent was obtained from all participants. The research protocols were approved by the Ethics Committee of Northern Ostrobotnia Hospital District, Finland.

Northern Finland Birth Cohort of 1966 (NFBC 1966)

The NFBC 1966 included 12,058 children born into the cohort, comprising 96% of all births during 1966 in the region (www.oulu.fi/nfbc).[13,14] Data collection in 1997 included clinical examination and serum sampling at age 31 for 6,007 individuals. Metabolomics data from this time point were used for the genetic analyses in the present study. Attendees in the 31-year field study (52%) were representative of the original cohort.[14] NMR-based metabolomics was measured from 5,709 individuals with serum sample available, of which 96% were fasting samples. Among these, 4,920 individuals had genotype information on rs12196 and rs17238484 variants in the *HMGCR* gene based on 370k Illumina HumanHap arrays.[13] Both common SNPs were imputed based on the 1000 Genome reference panel. Pregnant women were omitted from the genetic analyses. Informed written consent was obtained from all participants. The research protocols were approved by the Ethics Committee of Northern Ostrobotnia Hospital District, Finland.

FINRISK 1997

The FINRISK 1997 study was conducted to monitor the health of the Finnish population among persons aged 25–74 at recruitment (thl.fi/finriski).[3] In total, 8,444 individuals were recruited to represent the middle-aged population of five study locations across Finland. Participants completed questionnaires on use of lipid-lowering medication. Information on medication was complemented by national reimbursement records. Serum samples were stored at –70°C for later biomarker analyses. Samples were semi-fasting: participants were asked not to eat 4 hours prior to giving blood. The median fasting time was 5 h (interquartile range 4–6 h). NMR metabolomics was measured during 2012 for 7,610 participants with serum samples available. Among these, 4,599 individuals had information on rs12196 and rs17238484 variants in the *HMGCR* gene based on 670k Illumina HumanHap arrays after exclusion of pregnant women (n=48) and individuals on lipid-lowering medication (n=148). Both common SNPs were imputed based on the 1000 Genome reference panel. The study was approved by the Coordinating Ethical Committee of the Helsinki and Uusimaa Hospital District, Finland.

The Avon Longitudinal Study of Parents and Children (ALSPAC) – Children Cohort

The Avon Longitudinal Study of Parents and Children was established to understand how genetic and environmental characteristics influence health and development in parents and children (bristol.ac.uk/alspac).[9] Recruitment sought to enroll pregnant women in the Bristol area of the UK during 1990–92. Of the 14,541 pregnancies originally enrolled there were 14,062 live births of whom 13,988 were still alive at 12 months.[16] Additional recruitment of children who were potentially in the birth cohort has occurred since the children were aged 7 and this has resulted in a further 706 participants. All eligible participants have been invited to follow-up clinics since age 7 with recruitment rates between 45-70%. NMR-based metabolomics was measured from EDTA plasma samples collected during follow-up at age 7, 15, and 17. Genotype information on *HMGCR* variants were obtained from 610k Illumina HumanHap arrays generated by Sample Logistics and Genotyping Facilities at the Wellcome Trust Sanger Institute, UK, and LabCorp, USA. Genotype data were

available for n=2,456 adolescents with metabolomics data from the 17-year field survey (2009–2010). rs698912 was used as proxy for the validation variant rs17238484, linkage disequilibrium R^2=1. The study was approved by the ALSPAC Law and Ethics Committee and the UK National Health Service Research Ethics Committee. The main caregiver initially provided consent for child participation and from age 16-years the participants themselves have provided informed written consent.

British Women's Heart and Health Study (BWHHS)
The British Women's Heart and Health Study recruited 4,286 females between 1999 and 2001, who were randomly selected from 23 British towns and were aged between 60 and 79 years at assessment (lshtm.ac.uk/eph/ncde/research/bwhhs). Data collection including blood sampling took place between April 1999 and March 2001. Methods used have been described in detail previously.[17] Blood was drawn after a minimum of 8-hour fast and serum samples stored at −80°C. NMR metabolomics was measured for 3,777 serum samples available during 2012.[3] Among these, 3,030 study participants had information available on rs12196 variant in *HMGCR* and genomic population stratification based on the Illumina CardioMetabochip and were free of lipid-lowering medication at baseline (n=233 excluded). The SNP rs3761742 was used as proxy for the validation variant rs17238484 (linkage disequilibrium R^2=1). Local ethics committee approvals were obtained for the BWHHS and all women provided informed written consent.

Whitehall II Study (WHII)
The Whitehall II study was established in 1985 to investigate the importance of social class for health by following a cohort of 10,308 civil servants (men and women) initially aged 35–55 years working in the London offices of 20 Whitehall departments (ucl.ac.uk/whitehallII). Details of the study and methods used have been described previously.[18] NMR-based metabolomics was measured from 6,204 fasting serum samples collected during the phase-5 clinical examination in 1997–1999. Among these, 3,918 study participants had information available on rs12196 variant in *HMGCR* and genomic population stratification based on the Illumina IBC HumanCVD BeadChip and were free of lipid-lowering medication. Local ethics committee approvals were obtained for the Whitehall II Study and all participants provided informed written consent.

Cohort Acknowledgements
NFBC: Our most sincere thanks go to all cohort members and their parents. We would like to acknowledge the contribution of the late Academian of Science Leena Peltonen.

ALSPAC: We are extremely grateful to all the families who took part in this study, the midwives for their help in recruiting them, and the whole ALSPAC team, which includes interviewers, computer and laboratory technicians, clerical workers, research scientists, volunteers, managers, receptionists and nurses.

References
1. Tillin T, Hughes AD, Mayet J, et al. The relationship between metabolic risk factors and incident cardiovascular disease in Europeans, South Asians, and African Caribbeans. *J Am Coll Cardiol.* 2013;61:1777–86.
2. Tillin T, Forouhi NG, McKeigue PM, Chaturvedi N. Southall And Brent REvisited: cohort profile of SABRE, a UK population-based comparison of cardiovascular disease and diabetes in people of European, Indian Asian and African Caribbean origins. *Int J Epidemiol*. 2012;41:33–42.
3. Würtz P, Havulinna AS, Soininen P, et al. Metabolite profiling and cardiovascular event risk: a prospective study of three population-based cohorts. *Circulation*. 2015:131:774–85.

4. Mäntyselkä P, Kautiainen H, Saltevo J, et al. Weight change and lipoprotein particle concentration and particle size: a cohort study with 6.5-year follow-up. Atherosclerosis. 2012:223;239–43.
5. Würtz P, Tiainen M, Mäkinen VP, et al. Circulating metabolite predictors of glycemia in middle-aged men and women. Diabetes Care. 2012;35:1749–56.
6. Raitakari OT, Juonala M, Rönnemaa T, et al. Cohort profile: The cardiovascular risk in young Finns study. Int J Epidemiol. 2008:37;1220–6.
7. Nuotio J, Oikonen M, Magnussen CG, et al. Cardiovascular risk factors in 2011 and secular trends since 2007: the Cardiovascular Risk in Young Finns Study. Scand J Public Health. 2014;42:563–71.
8. Würtz P, Wang Q, Kangas AJ, et al. Metabolic signatures of adiposity: Mendelian randomization analysis and effects of weight change. PLoS Med. 2014;11:e1001765.
9. Fraser A, Macdonald-Wallis C, Tilling K, et al. Cohort profile: the Avon Longitudinal Study of Parents and Children: ALSPAC mothers cohort. Int J Epidemiol. 2013;42:97–110.
10. Järvelin MR, Hartikainen-Sorri AL, Rantakallio P. Labour induction policy in hospitals of different levels of specialisation. Br J Obstet Gynaecol. 1993;100:310-5.
11. Kantomaa MT, Stamatakis E, Kankaanpää A, et al. Physical activity and obesity mediate the association between childhood motor function and adolescents' academic achievement. Proc Natl Acad Sci USA. 2013;110:1917–22.
12. Veltsista A, Laitinen J, Sovio U, Roma E, Järvelin MR, Bakoula C. Relationship between eating behavior, breakfast consumption, and obesity among Finnish and Greek adolescents. J Nutr Educ Behav. 2010;42:417–21.
13. Sabatti C, Service SK, Hartikainen AL, et al. Genome-wide association analysis of metabolic traits in a birth cohort from a founder population. Nat Genet. 2009;41: 35–46.
14. Järvelin MR, Sovio U, King V, et al. Early life factors and blood pressure at age 31 years in the 1966 northern Finland birth cohort. Hypertension. 2004;44:838–46.
15. Vartiainen E, Laatikainen T, Peltonen M, et al. Thirty-five-year trends in cardiovascular risk factors in Finland. Int J Epidemiol. 2010;39:504–18.
16. Boyd A, Golding J, Macleod J, et al. Cohort Profile: The Avon Longitudinal Study of Parents and Children: ALSPAC mothers cohort. Int J Epidemiol. 2013;42:111–27.
17. Lawlor DA, Bedford C, Taylor M, Ebrahim S. Geographical variation in cardiovascular disease, risk factors, and their control in older women: British Women's Heart and Health Study. J Epidemiol Community Health 2003;57:134–40.
18. Marmot M, Brunner E. Cohort Profile: The Whitehall II study. Int J Epidemiol. 2005;34:251-256.
19. Soininen P, Kangas AJ, Würtz P, Suna T, Ala-Korpela M. Quantitative serum nuclear magnetic resonance metabolomics in cardiovascular epidemiology and genetics. Circ Cardiovasc Genet. 2015;8:192–206.
20. Würtz P, Kangas AJ, Soininen P, et al. Lipoprotein subclass profiling reveals pleiotropy in the genetic variants of lipid risk factors for coronary heart disease: a note on Mendelian randomization studies. J Am Coll Cardiol. 2013;62:1906–8.

Supplementary Table 1*. Mean absolute concentrations of lipoprotein, fatty acid and metabolite measures in the study population and absolute concentration changes associated with starting statin therapy and rs12916 in *HMGCR.

Metabolic measure	Mean (SD)	Absolute concentration change associated with starting statins<br><br>Beta [95%CI]<br>(meta-analysis of 716 statin starters and 4,874 non-users)	Absolute difference per T-allele of rs12196 in *HMGCR*<br><br>Beta [95%CI]<br>(meta-analysis of n=27,914)
**Lipoprotein subclass particle concentrations**			
Extremely large VLDL (μmol/L)	0.00011 (0.00012)	-0.000041 [-0.000053, -0.000029]	-0.0000022 [-0.0000041, -0.0000004]
Very large VLDL (μmol/L)	0.00057 (0.00063)	-0.00012 [-0.00019, -0.00005]	-0.000012 [-0.000021, -0.000003]
Large VLDL (μmol/L)	0.0034 (0.0032)	-0.00056 [-0.00093, -0.00018]	-0.000057 [-0.00011, -0.000009]
Medium VLDL (μmol/L)	0.013 (0.008)	-0.0023 [-0.0033, -0.0014]	-0.00021 [-0.00034, -0.00009]
Small VLDL (μmol/L)	0.028 (0.010)	-0.0059 [-0.0069, -0.0049]	-0.00041 [-0.00057, -0.00025]
Very small VLDL (μmol/L)	0.039 (0.010)	-0.012 [-0.013, -0.011]	-0.0006 [-0.0008, -0.0004]
IDL (μmol/L)	0.099 (0.023)	-0.038 [-0.040, -0.035]	-0.0019 [-0.0023, -0.0015]
Large LDL (μmol/L)	0.16 (0.04)	-0.065 [-0.068, -0.061]	-0.0034 [-0.0040, -0.0027]
Medium LDL (μmol/L)	0.13 (0.03)	-0.053 [-0.057, -0.050]	-0.0028 [-0.0033, -0.0022]
Small LDL (μmol/L)	0.15 (0.04)	-0.058 [-0.062, -0.054]	-0.003 [-0.004, -0.002]
Very large HDL (μmol/L)	0.39 (0.18)	-0.063 [-0.079, -0.048]	-0.0014 [-0.0042, 0.0014]
Large HDL (μmol/L)	1.0 (0.4)	0.043 [0.007, 0.080]	0.005 [-0.001, 0.011]
Medium HDL (μmol/L)	1.8 (0.3)	0.036 [-0.004, 0.075]	0.000031 [-0.0058, 0.0059]
Small HDL (μmol/L)	4.3 (0.5)	0.099 [0.044, 0.153]	0.00085 [-0.0071, 0.0088]
**Cholesterol (C)**			
Total C (mmol/L)	4.7 (0.9)	-1.4 [-1.4, -1.3]	-0.077 [-0.092, -0.061]
Non-HDL C (mmol/L)	3.2 (0.9)	-1.3 [-1.4, -1.2]	-0.076 [-0.090, -0.061]
Remnant C (mmol/L)	1.5 (0.4)	-0.51 [-0.54, -0.47]	-0.03 [-0.04, -0.02]
VLDL C (mmol/L)	0.73 (0.24)	-0.22 [-0.24, -0.19]	-0.014 [-0.018, -0.010]
IDL C (mmol/L)	0.74 (0.18)	-0.29 [-0.31, -0.28]	-0.016 [-0.019, -0.013]
LDL C (mmol/L)	1.7 (0.5)	-0.81 [-0.86, -0.77]	-0.046 [-0.054, -0.037]
HDL C (mmol/L)	1.5 (0.3)	-0.02 [-0.05, 0.01]	0.00036 [-0.00502, 0.00575]
HDL_2 C (mmol/L)	0.95 (0.31)	0.0017 [-0.025, 0.028]	0.00096 [-0.0037, 0.0056]
HDL_3 C (mmol/L)	0.52 (0.04)	-0.022 [-0.027, -0.018]	-0.0014 [-0.0023, -0.0006]
Esterified C (mmol/L)	3.4 (0.7)	-0.97 [-1.03, -0.90]	-0.053 [-0.064, -0.041]
Free C (mmol/L)	1.3 (0.3)	-0.39 [-0.42, -0.37]	-0.021 [-0.026, -0.017]
Esterified C (%)	72 (2)	0.081 [-0.138, 0.299]	0.00016 [-0.00049, 0.00082]
**Triglycerides (TG)**			
Total TG (mmol/L)	1.2 (0.5)	-0.20 [-0.26, -0.15]	-0.017 [-0.025, -0.008]
VLDL TG (mmol/L)	0.72 (0.43)	-0.095 [-0.143, -0.047]	-0.011 [-0.017, -0.004]
IDL TG (mmol/L)	0.12 (0.03)	-0.031 [-0.034, -0.027]	-0.0016 [-0.0021, -0.0010]
LDL TG (mmol/L)	0.21 (0.07)	-0.054 [-0.061, -0.048]	-0.0033 [-0.0044, -0.0021]

HDL TG (mmol/L)	0.12 (0.04)	-0.017 [-0.021, -0.013]	-0.00067 [-0.0013, -0.00002]
**Phospholipids (PL)**			
Total PL (mmol/L)	3.0 (0.4)	-0.41 [-0.46, -0.37]	-0.024 [-0.032, -0.017]
VLDL PL (mmol/L)	0.45 (0.17)	-0.12 [-0.14, -0.10]	-0.0082 [-0.011, -0.0054]
IDL PL (mmol/L)	0.31 (0.07)	-0.12 [-0.12, -0.11]	-0.0064 [-0.0076, -0.0052]
LDL PL (mmol/L)	0.67 (0.14)	-0.22 [-0.23, -0.20]	-0.012 [-0.014, -0.009]
HDL PL (mmol/L)	1.6 (0.3)	0.039 [0.010, 0.068]	0.0021 [-0.0032, 0.0074]
Cholines (mmol/L)	2.0 (0.4)	-0.30 [-0.33, -0.26]	-0.015 [-0.021, -0.008]
Sphingomyelin (mmol/L)	0.38 (0.06)	-0.052 [-0.059, -0.045]	-0.0038 [-0.0052, -0.0023]
Phosphoglycerides (mmol/L)	1.3 (0.3)	-0.18 [-0.21, -0.15]	-0.011 [-0.016, -0.006]
**Lipoprotein particle size**			
VLDL particle size (nm)	36 (1)	0.26 [0.13, 0.38]	-0.0063 [-0.026, 0.014]
LDL particle size (nm)	24 (0)	0.0054 [-0.013, 0.024]	0.001 [-0.001, 0.003]
HDL particle size (nm)	10 (0)	-0.029 [-0.048, -0.009]	-0.000063 [-0.0037, 0.0036]
**Apolipoproteins**			
Apolipoprotein B (g/L)	0.91 (0.21)	-0.27 [-0.29, -0.25]	-0.015 [-0.019, -0.012]
Apolipoprotein A-I (g/L)	1.6 (0.2)	-0.08 [-0.10, -0.06]	-0.005 [-0.008, -0.002]
**Fatty acids**			
Total fatty acids (mmol/L)	11 (2)	-2.0 [-2.2, -1.7]	-0.11 [-0.16, -0.07]
Saturated fatty acids (mmol/L)	3.8 (1.0)	-0.69 [-0.79, -0.59]	-0.038 [-0.055, -0.021]
MUFA (mmol/L)	3.0 (0.9)	-0.41 [-0.49, -0.32]	-0.026 [-0.041, -0.010]
PUFA (mmol/L)	4.0 (0.8)	-0.81 [-0.89, -0.73]	-0.049 [-0.062, -0.035]
Omega-6 fatty acids (mmol/L)	3.6 (0.7)	-0.74 [-0.81, -0.67]	-0.044 [-0.056, -0.032]
Linoleic acid (mmol/L)	3.0 (0.6)	-0.74 [-0.81, -0.68]	-0.043 [-0.053, -0.033]
Omega-3 fatty acids (mmol/L)	0.43 (0.14)	-0.056 [-0.072, -0.041]	-0.0045 [-0.0067, -0.0023]
Docosahexaenoic acid (mmol/L)	0.17 (0.06)	-0.015 [-0.020, -0.009]	-0.0012 [-0.0022, -0.0003]
**Fatty acid ratios**			
Saturated fatty acids (%)	35 (2)	0.083 [-0.230, 0.40]	0.042 [0.008, 0.075]
MUFA (%)	27 (3)	0.77 [0.42, 1.12]	0.0082 [-0.044, 0.061]
PUFA (%)	38 (4)	-0.80 [-1.24, -0.36]	-0.05 [-0.11, 0.01]
Omega-6 fatty acids (%)	34 (4)	-0.89 [-1.31, -0.46]	-0.049 [-0.101, 0.004]
Linoleic acid (%)	28 (4)	-1.8 [-2.2, -1.4]	-0.10 [-0.16, -0.05]
Omega-3 fatty acids (%)	4.0 (1.0)	0.26 [0.14, 0.39]	-0.0012 [-0.016, 0.014]
Docosahexaenoic acid (%)	1.6 (0.5)	0.15 [0.10, 0.20]	0.0041 [-0.0029, 0.011]
Degree of unsaturation	1.2 (0.1)	0.0076 [-0.0018, 0.017]	0.00056 [-0.00065, 0.0018]
**Amino acids**			
Alanine (µmol/L)	358 (64)	6.3 [-0.5, 13.2]	0.043 [-1.028, 1.113]
Glutamine (µmol/L)	516 (84)	4.9 [-3.5, 13.4]	0.62 [-0.57, 1.81]
Glycine (µmol/L)	308 (62)	-2.4 [-8.1, 3.3]	0.10 [-0.96, 1.16]
Branched-chain amino acids			
Isoleucine (µmol/L)	52 (15)	-2.2 [-3.5, -0.8]	-0.16 [-0.40, 0.08]
Leucine (µmol/L)	81 (19)	-1.1 [-2.8, 0.7]	-0.042 [-0.281, 0.197]
Valine (µmol/L)	187 (37)	-2.6 [-6.3, 1.0]	0.15 [-0.45, 0.75]
Aromatic amino acids			

Phenylalanine (µmol/L)	75 (12)	-2.4 [-3.7, -1.1]	-0.022 [-0.188, 0.145]
Tyrosine (µmol/L)	56 (12)	-1.1 [-2.3, 0.0]	0.042 [-0.154, 0.237]
Histidine (µmol/L)	69 (13)	-1.8 [-3.2, -0.4]	0.023 [-0.161, 0.207]
**Glycolysis metabolites**			
Glucose (mmol/L)	4.6 (0.9)	0.065 [-0.024, 0.154]	0.01 [0.00, 0.02]
Lactate (mmol/L)	1.4 (0.4)	0.034 [-0.022, 0.090]	-0.0048 [-0.012, 0.0018]
Pyruvate (µmol/L)	79 (26)	5.5 [2.3, 8.8]	0.076 [-0.36, 0.51]
Citrate (µmol/L)	102 (20)	-0.074 [-2.45, 2.30]	0.052 [-0.33, 0.43]
Glycerol (µmol/L)	94 (33)	1.5 [-2.9, 5.9]	-0.64 [-1.32, 0.05]
**Ketone bodies**			
Acetoacetate (µmol/L)	41 (31)	0.20 [-4.87, 5.27]	0.10 [-0.55, 0.75]
Beta-hydroxybutyrate (µmol/L)	105 (73)	-9.5 [-20.1, 1.2]	-0.39 [-2.21, 1.42]
**Miscellaneous**			
Creatinine (µmol/L)	62 (11)	-0.54 [-1.68, 0.61]	0.25 [0.07,0.42]
Albumin (cu)	0.094 (0.007)	-0.00059 [-0.0013, 0.00016]	-0.00014 [-0.00025, -0.00002]
Acetate (µmol/L)	57 (21)	-3.4 [-5.4, -1.3]	-0.13 [-0.35, 0.10]
**Inflammation**			
Glycoprotein acetyls (mmol/L)	1.4 (0.2)	-0.037 [-0.057, -0.017]	-0.0032 [-0.0067, 0.0004]

Mean concentrations of the metabolic measures and standard deviations (SD) were averaged across the four longitudinal cohorts. Absolute concentration changes associated with starting statin therapy are indicated, corresponding to the SD-scaled values shown in the left columns of Figures 1–3. The associations of rs12916-T allele with the metabolic measures in absolute concentration units are also indicated, corresponding to the SD-scaled values in the right columns of Figures 1–3.

cu: standardized concentration units.

All the 80 lipoprotein, lipid and metabolite measures were quantified using the same high-throughput serum NMR metabolomics platform.[19] The 14 lipoprotein subclass sizes were defined as follows: extremely large VLDL with particle diameters from 75 nm upwards and a possible contribution of chylomicrons, five VLDL subclasses (average particle diameters of 64.0 nm, 53.6 nm, 44.5 nm, 36.8 nm, and 31.3 nm), IDL (28.6 nm), three LDL subclasses (25.5 nm, 23.0 nm, and 18.7 nm), and four HDL subclasses (14.3 nm, 12.1 nm, 10.9 nm, and 8.7 nm). The mean size for VLDL, LDL and HDL particles was calculated by weighting the corresponding subclass diameters with their particle concentrations.

Remnant cholesterol was defined as VLDL-cholesterol + IDL-cholesterol, which is equivalent to total-cholesterol - HDL-cholesterol - LDL-cholesterol.[20]

***Supplementary Table 2*. Clinical characteristics of the eight study populations used for the genetic analyses stratified by rs12916 genotype.**

**Characteristics**	**Avon Longitudinal Study of Parents and Children: ALSPAC children n=2,456**			**Avon Longitudinal Study of Parents and Children: ALSPAC mothers n=3,137**			**British Women's Heart and Health Study (BWHHS) n=3,030**		
Genotype	C/C	C/T	T/T	C/C	C/T	T/T	C/C	C/T	T/T
Number of individuals	338	1192	926	456	1481	1200	483	1459	1088
Male [%]	55	50	52	0	0	0	0	0	0
Age [year]	17.8 (0.4)	17.8 (0.4)	17.8 (0.4)	47.9 (4.2)	47.8 (4.3)	48.0 (4.3)	68.3 (5.4)	69.0 (5.5)	68.8 (5.7)
BMI [kg/m2]	22.6 (3.6)	22.6 (3.9)	22.8 (3.9)	26.4 (4.8)	26.4 (5.0)	26.4 (5.1)	27.3 (5.1)	27.3 (4.8)	27.8 (5.1)
Systolic blood pressure [mmHg]	114 (10)	115 (10)	115 (10)	118 (12)	118 (12)	118 (12)	145 (24)	148 (25)	147 (25)
Plasma glucose [mmol/L]	5.0 [4.7,5.3]	5.0 [4.8,5.2]	5.0 [4.8,5.3]	5.2 [4.9,5.5]	5.1 [4.9,5.4]	5.2 [4.9,5.4]	5.8 [5.4,6.1]	5.7 [5.4,6.1]	5.7 [5.4,6.1]
Triglycerides [mmol/L]	0.8 [0.6,1.0]	0.8 [0.6,1.0]	0.8 [0.6,1.0]	0.9 [0.7,1.2]	0.9 [0.7,1.2]	0.9 [0.7,1.1]	1.5 [1.2,2.2]	1.6 [1.2,2.2]	1.6 [1.2,2.3]
Total cholesterol [mmol/L]	3.9 (0.7)	3.8 (0.7)	3.7 (0.7)	5.0 (0.9)	5.0 (0.8)	4.9 (0.9)	6.8 (1.2)	6.7 (1.2)	6.6 (1.1)
HDL cholesterol [mmol/L]	1.3 (0.3)	1.3 (0.3)	1.3 (0.3)	1.5 (0.4)	1.5 (0.4)	1.5 (0.4)	1.7 (0.5)	1.7 (0.5)	1.7 (0.4)
Friedewald LDL cholesterol [mmol/L]	2.2 (0.6)	2.1 (0.6)	2.0 (0.6)	3.0 (0.8)	3.0 (0.8)	2.9 (0.8)	4.3 (1.1)	4.2 (1.1)	4.2 (1.0)
rs12916 minor allele frequency (C)	43%			40%			40%		
LDL cholesterol per rs12916-T allele, beta [95%CI] in mmol/L; Variance explained	-0.11 [-0.17, -0.048] R^2=0.0051			-0.11 [-0.16, -0.055] R^2=0.0052			-0.058 [-0.11, -0.006] R^2=0.0016		

Values are mean (SD) and median [interquartile range] for normally distributed and skewed variables, respectively. P-value of Hardy-Weinberg equilibrium for rs12196 in *HMGCR* was P>0.01 in all cohorts.

Supplementary Table 2 (Continued)

Characteristics	Cardiovascular Risk in Young Finns Study (YFS) n=1,905			FINRISK 1997 n=4,403			Northern Finland Birth Cohort of 1986 (NFBC-1986) n=4,145		
Genotypes	C/C	C/T	T/T	C/C	C/T	T/T	C/C	C/T	T/T
Number of individuals	362	986	557	856	2221	1326	823	1965	1357
Male [%]	44	45	48	47	48	49	50	49	51
Age [year]	37.6 (4.8)	37.7 (5.2)	37.7 (4.8)	47.2 (13.5)	47.1 (12.9)	47.0 (13.2)	16.1 (0.4)	16.0 (0.3)	16.1 (0.4)
BMI [kg/m2]	25.6 (4.7)	26.0 (4.7)	26.1 (4.8)	26.6 (4.4)	26.5 (4.5)	26.5 (4.5)	21.4 (3.5)	21.2 (3.5)	21.1 (3.2)
Systolic blood pressure [mmHg]	121 (15)	121 (14)	120 (14)	135 (19)	135 (20)	135 (20)	116 (13)	115 (13)	116 (13)
Plasma glucose [mmol/L]	5.2 [4.9,5.6]	5.2 [4.9,5.6]	5.2 [4.9,5.6]	5.0 [4.7,5.3]	5.0 [4.7,5.3]	5.0 [4.6,5.3]	5.1 [4.9,5.4]	5.2 [4.9,5.4]	5.1 [4.9,5.4]
Triglycerides [mmol/L]	1.1 [0.8,1.6]	1.1 [0.8,1.6]	1.1 [0.8,1.7]	1.2 [0.9,1.7]	1.2 [0.9,1.7]	1.2 [0.8,1.7]	0.8 [0.6,1.0]	0.7 [0.6,1.0]	0.7 [0.6,1.0]
Total cholesterol [mmol/L]	5.1 (0.9)	5.1 (0.9)	4.9 (0.8)	5.6 (1.1)	5.6 (1.0)	5.4 (1.0)	4.3 (0.8)	4.3 (0.8)	4.2 (0.8)
HDL cholesterol [mmol/L]	1.3 (0.3)	1.3 (0.3)	1.3 (0.3)	1.4 (0.3)	1.4 (0.4)	1.4 (0.3)	1.4 (0.3)	1.4 (0.3)	1.4 (0.3)
Friedewald LDL cholesterol [mmol/L]	3.1 (0.8)	3.1 (0.8)	2.9 (0.7)	3.6 (0.9)	3.5 (0.9)	3.4 (0.9)	2.5 (0.7)	2.5 (0.7)	2.4 (0.7)
rs12916 minor allele frequency (C)	47%			45%			44%		
LDL cholesterol per rs12916-T allele, beta [95%CI] in mmol/L; Variance explained	-0.14 [-0.20, -0.073]; R^2=0.0090			-0.12 [-0.16, -0.075]; R^2=0.0067			-0.095 [-0.14, -0.052]; R^2=0.0046		

Values are mean (SD) and median [interquartile range] for normally distributed and skewed variables, respectively. P-value of Hardy-Weinberg equilibrium for rs12196 in *HMGCR* was P>0.01 in all cohorts.

Supplementary Table 2 (Continued)

Characteristics	Northern Finland Birth Cohort of 1966 (NFBC-1966) n=4,920			Whitehall II study (WHII) n=3,918		
Genotypes	C/C	C/T	T/T	C/C	C/T	T/T
Number of individuals	936	2432	1552	631	1886	1401
Male [%]	51	50	50	77	73	74
Age [year]	31.1 (0.4)	31.1 (0.3)	31.2 (0.4)	55.2 (6.0)	55.3 (6.0)	55.3 (5.8)
BMI [kg/m2]	24.6 (4.1)	24.6 (4.1)	24.6 (4.2)	25.8 (3.7)	26.0 (3.9)	26.0 (3.8)
Systolic blood pressure [mmHg]	125 (14)	125 (13)	125 (13)	123 (16)	122 (16)	122 (16)
Plasma glucose [mmol/L]	5.0 [4.8,5.3]	5.0 [4.7,5.3]	5.0 [4.7,5.3]	5.1 [4.7,5.4]	5.0 [4.7,5.4]	5.0 [4.7,5.3]
Triglycerides [mmol/L]	1.0 [0.7,1.4]	1.0 [0.7,1.4]	1.0 [0.7,1.4]	1.1 [0.8,1.6]	1.1 [0.8,1.6]	1.1 [0.8,1.6]
Total cholesterol [mmol/L]	5.1 (1.0)	5.1 (1.0)	5.0 (1.0)	6.0 (1.0)	5.9 (1.0)	5.8 (1.0)
HDL cholesterol [mmol/L]	1.5 (0.4)	1.5 (0.4)	1.6 (0.4)	1.5 (0.4)	1.5 (0.4)	1.5 (0.4)
Friedewald LDL cholesterol [mmol/L]	3.0 (0.9)	3.0 (0.9)	2.9 (0.9)	4.0 (0.9)	3.9 (0.9)	3.8 (0.9)
rs12916 minor allele frequency (C)	45%			40%		
LDL cholesterol per rs12916-T allele, beta [95%CI] in mmol/L; Variance explained	-0.071 [-0.11, -0.031]; R^2=0.0025			-0.097 [-0.14, -0.052]; R^2=0.0045		

Values are mean (SD) and median [interquartile range] for normally distributed and skewed variables, respectively. P-value of Hardy-Weinberg equilibrium for rs12196 in *HMGCR* was P>0.01 in all cohorts.

Supplementary Figure 1. Representative NMR spectra before and after statin use.

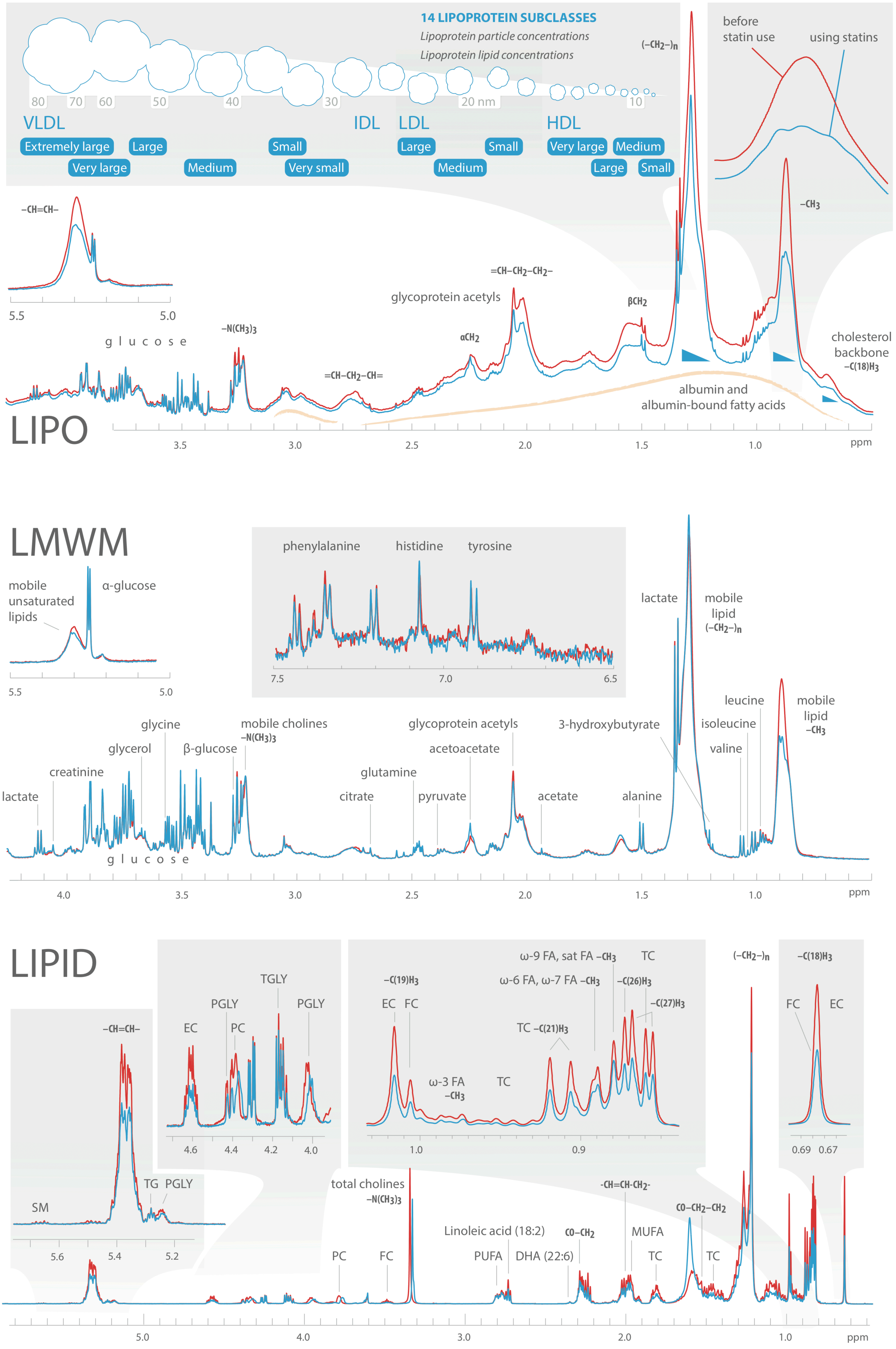

Example of the three NMR spectral windows (LIPO, LMWM and LIPID) measured by the high-throughput serum metabolomics platform.[19] The spectral data are shown for a single individual before and after starting statin therapy. An individual with a large absolute change in LDL-C (-2.6 mmol/L) was chosen to visually emphasize effects of statin use on the spectral characteristics. The figure is meant only to exemplify the overall characteristics of the NMR spectral data and illustrate the approximate molecular content as well as signal assignments. All statistical data analyses were conducted based on the lipoprotein, lipid and metabolite measures quantified in absolute concentration units. No direct analysis of statin effects on the spectral data were performed — the metabolic biomarker data were analyzed in the present study similarly as for any other quantitative clinical chemistry measure.

The LIPO window is acquired from a native serum sample. It is dominated by broad signals arising from macromolecules, mainly lipoprotein lipids and albumin. Despite the broad overall characteristics and substantial overlap of the resonances, appropriate data analyses provide abundant information on lipoprotein particles as indicated by the inset illustrating the lipoprotein subclasses. The particle sizes range from diameters of ~8 nm for the smallest HDL particles to >80 nm for the largest VLDL particles. The LMWM window is also acquired from the native serum sample using a special NMR pulse sequence, which suppresses most of the broad macromolecule and lipoprotein lipid signals. This enhances the detection and quantification of rapidly tumbling smaller solutes. The LMWM spectrum shows signals from various low-molecular-weight molecules as well as some lipid resonances representing the most mobile lipid molecules in the lipoprotein particles. The LIPID window is run separately from serum lipid extracts; the extraction procedure breaks down the lipoprotein particles yielding information on the individual lipid species inside the particles.

The spectra are shown in red for the measurement before statin use and in blue for the measurement after initiation of statin therapy. The large decrease in LDL-C (-2.6 mmol/L) is evident from all the key lipid resonances between 0.5 and 3.2 ppm in the LIPO spectrum; the $–CH_3$ resonance is shown in an inset in the top right-hand corner of the figure. The lipids in the IDL and LDL subclasses give rise to their most pronounced resonances in the middle of the $–CH_3$ peak where the largest difference between the spectra are apparent. For this person, also serum triglycerides decreased (-0.7 mmol/L) after starting statin treatment; this is also evident from the lipid resonances, e.g., on the left-hand side of the $–CH_3$ peak, where the triglyceride-rich VLDL subclasses give rise to their main spectral contribution. In accordance with the overall results shown in Figure 3, there are very little differences between the spectra in the LMWM window. The only exception hereof is a reflection of the decrease in the mobile lipids, mainly VLDL-related triglycerides. In contrast hereto, marked differences are observed throughout the LIPID window. These reflect the overall results shown in Figure 2, namely that the absolute concentrations of all fatty acid measures decrease along with starting statin therapy. This is a direct consequence of large decreases in lipoprotein cholesterol and triglyceride concentrations.

Abbreviations: EC, esterified cholesterol; FA, fatty acid; FC, free cholesterol; HDL, high-density lipoprotein; IDL, intermediate-density lipoprotein; LDL, low-density lipoprotein; MUFA, monounsaturated fatty acids; PC, phosphatidylcholine; PGLY, phosphoglycerides; PUFA, polyunsaturated fatty acids; sat, saturated; SM, sphingomyelin; TC, total serum cholesterol; TG, total serum triglycerides; TGLY, TG backbone; VLDL, very-low-density lipoprotein.

Supplementary Figure 2. Lipid and metabolite changes associated with starting statin therapy relative to baseline concentrations.

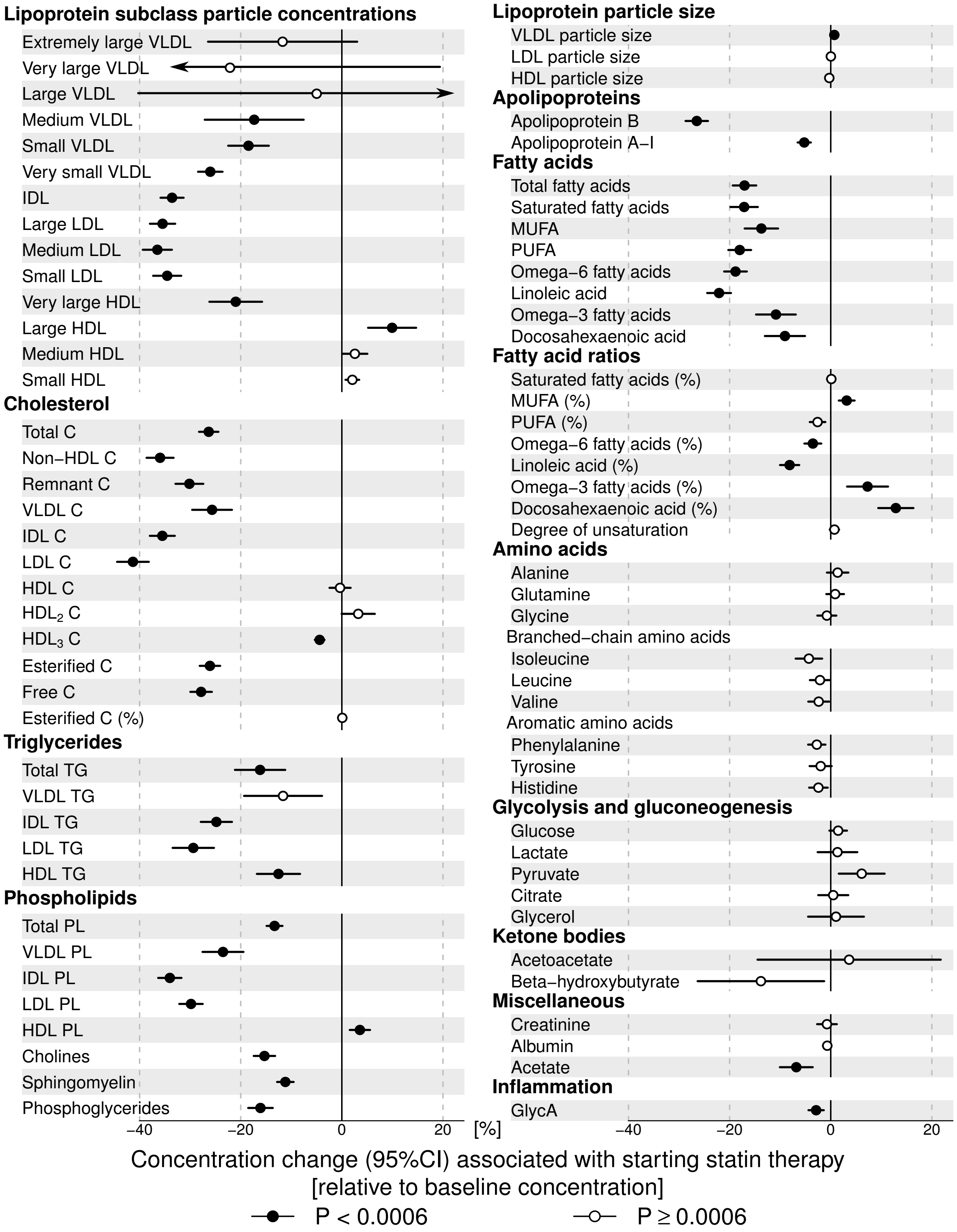

Lipoprotein, fatty acid and metabolite concentration changes associated with starting statin therapy in percentage change relative to baseline concentrations. The relative concentration changes were assessed in each of the four longitudinal cohorts with adjustment for age and sex, and subsequently meta-analyzed. Error bars denote 95% confidence intervals. Circles without visible error bars indicate that the confidence intervals are within the symbols. Wide confidence intervals arise for some metabolic measures with very low concentrations and highly skewed distributions.

Supplementary Figure 3A-F. Lipid and metabolite changes associated with starting statin therapy and rs12196 in *HMGCR* separately for the individual cohorts.

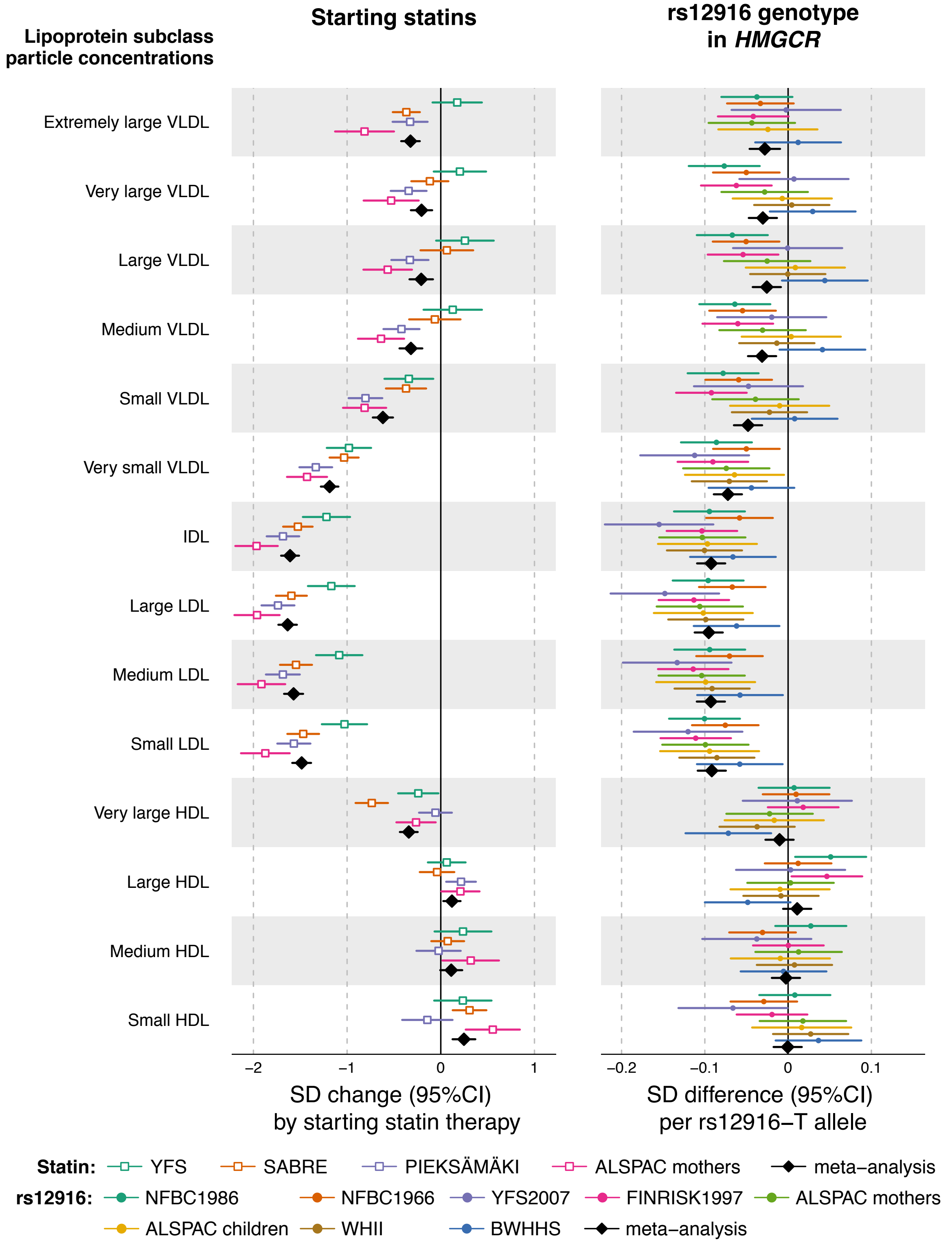

Supplementary Figure 3A. Left: Lipoprotein changes associated with starting statin therapy in the four cohorts with profiling at two time-points. Associations were adjusted for age and sex. Results are shown in SD-scaled concentration units.
Right: Lipoprotein associations with rs12196 in *HMGCR* in the eight individual cohorts. Associations were adjusted for age, sex, and population stratification. Results are shown in SD-scaled concentration units.

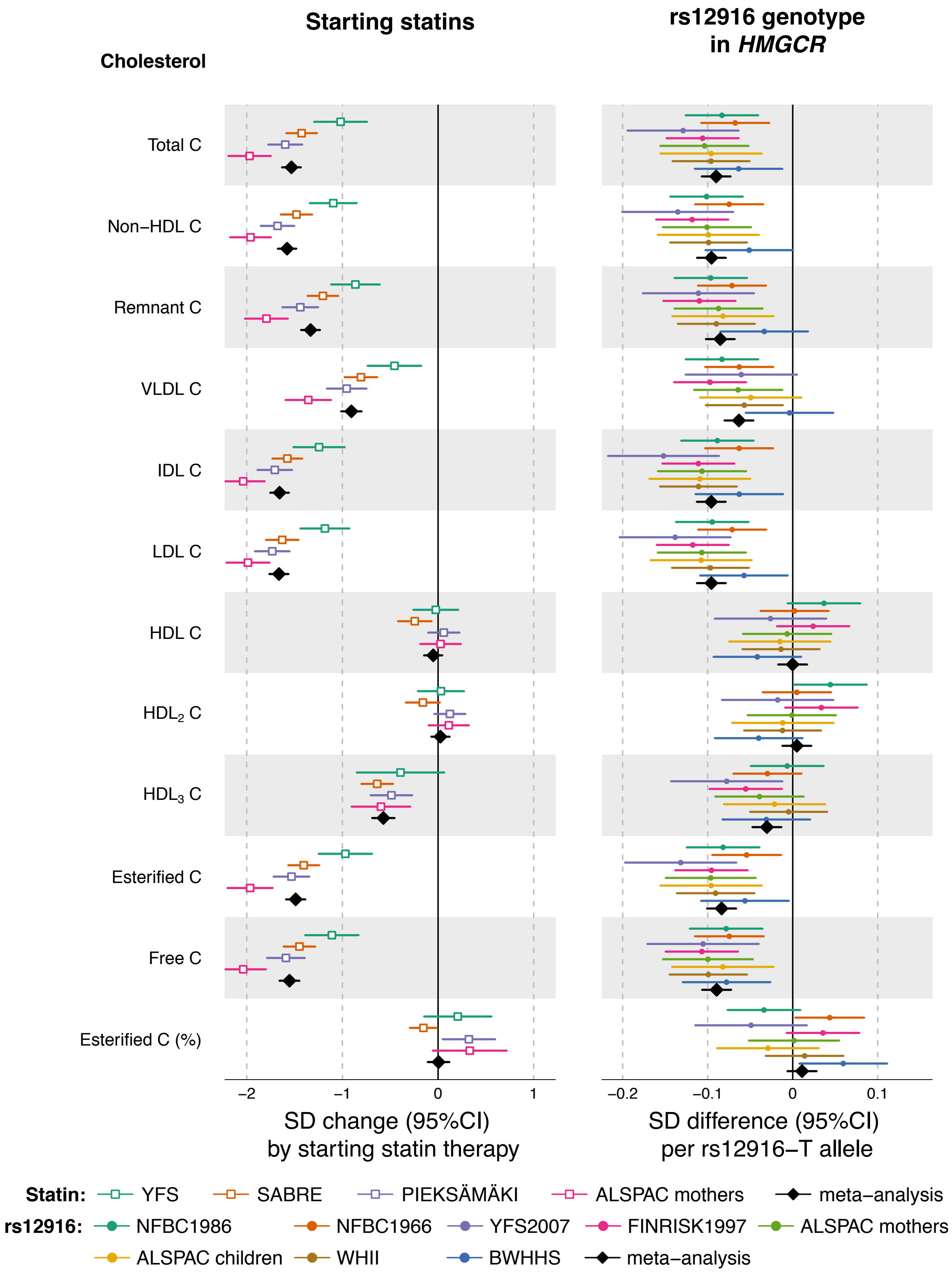

Supplementary Figure 3B. Left: Lipoprotein lipid changes associated with starting statin therapy in the four cohorts with profiling at two time-points. Associations were adjusted for age and sex. Results are shown in SD-scaled concentration units.

Right: Lipoprotein lipid associations with rs12196 in *HMGCR* in the eight individual cohorts. Associations were adjusted for age, sex, and population stratification. Results are shown in SD-scaled concentration units.

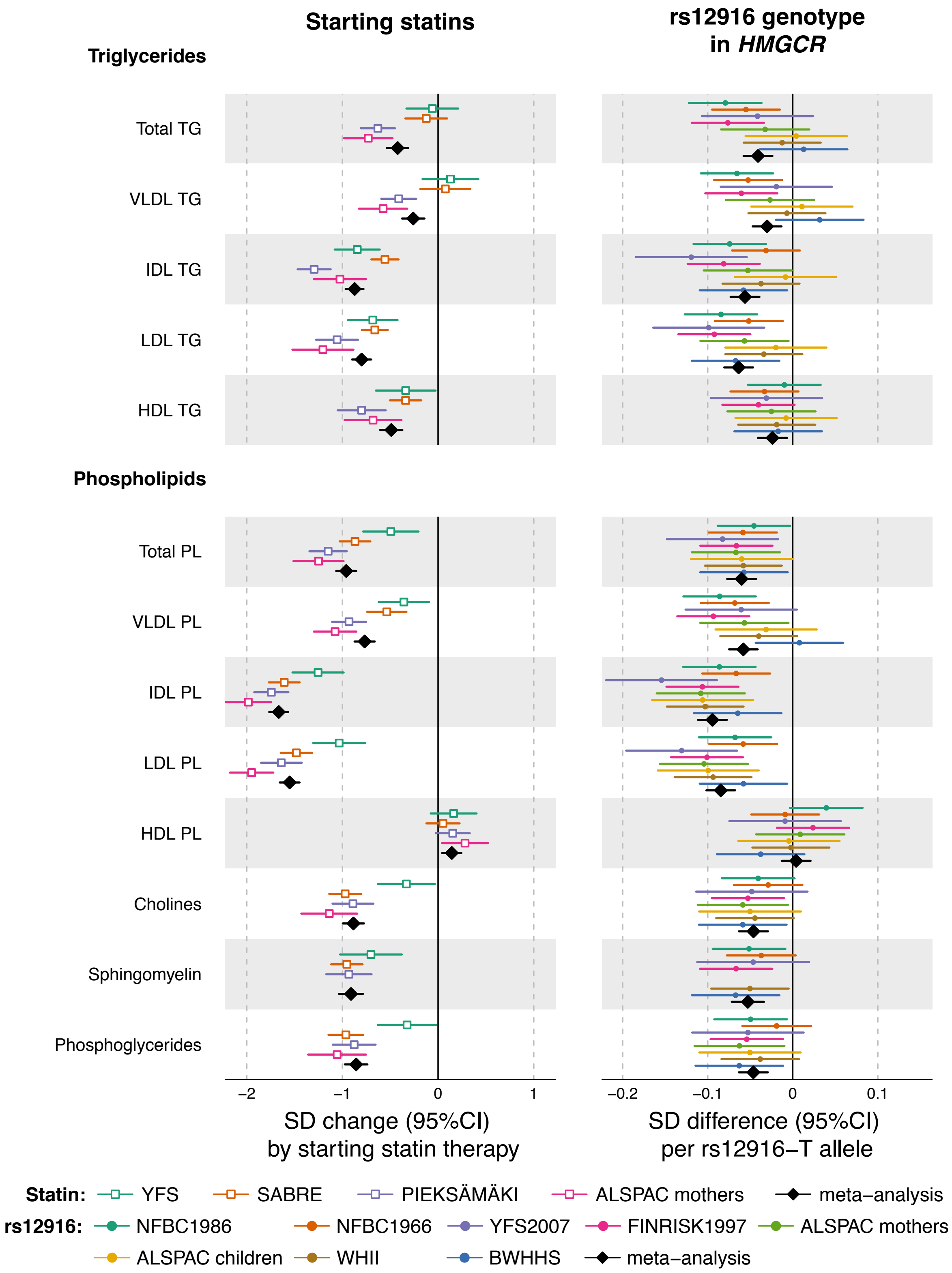

Supplementary Figure 3C. Left: Lipoprotein lipid changes associated with starting statin therapy in the four cohorts with profiling at two time-points. Associations were adjusted for age and sex. Results are shown in SD-scaled concentration units.

Right: Lipoprotein lipid associations with rs12196 in *HMGCR* in the eight individual cohorts. Associations were adjusted for age, sex, and population stratification. Results are shown in SD-scaled concentration units.

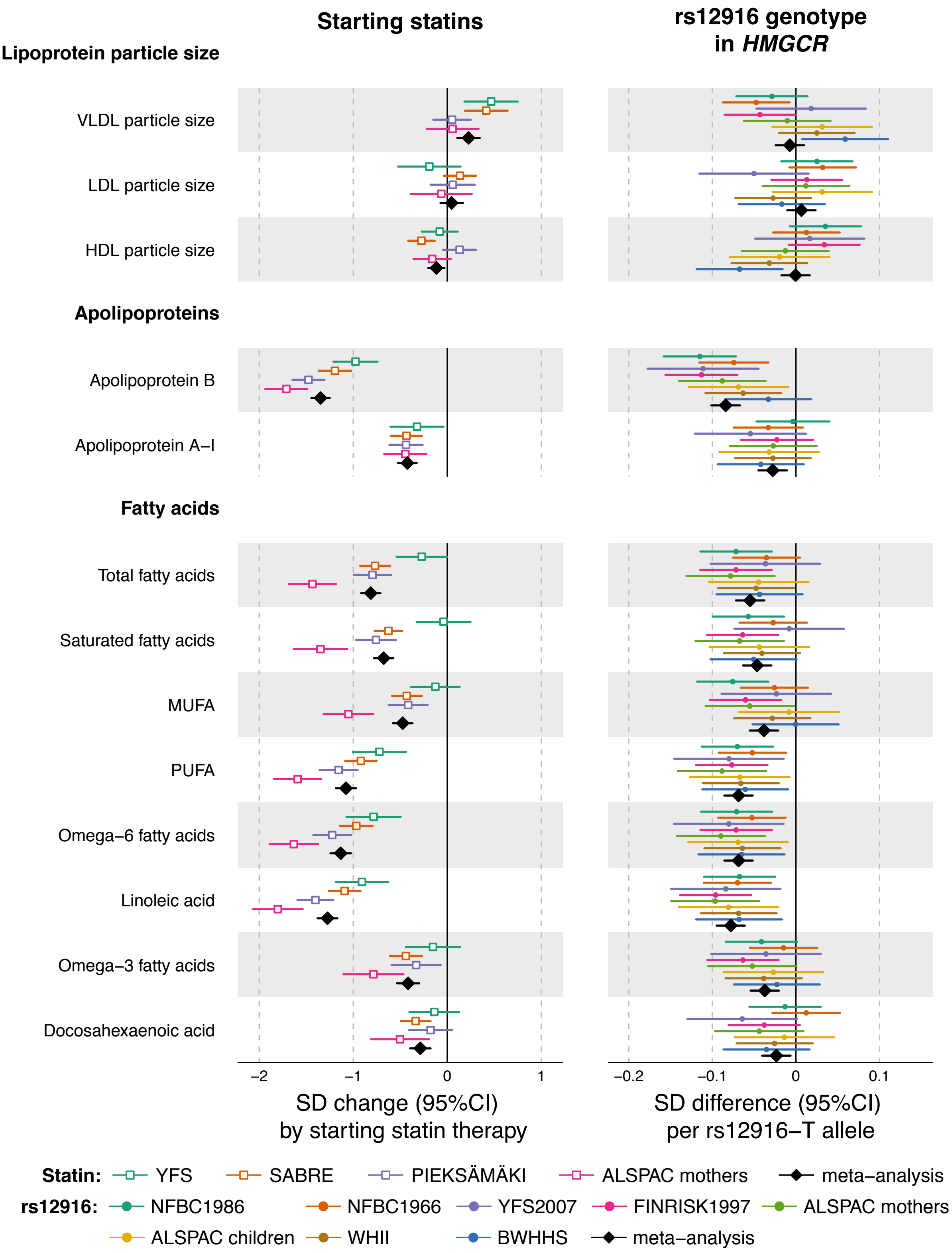

Supplementary Figure 3D. Left: Lipoprotein and fatty acid changes associated with starting statin therapy in the four cohorts with profiling at two time-points. Associations were adjusted for age and sex. Results are shown in SD-scaled concentration units.

Right: Lipoprotein and fatty acid associations with rs12196 in *HMGCR* in the eight individual cohorts. Associations were adjusted for age, sex, and population stratification. Results are shown in SD-scaled concentration units.

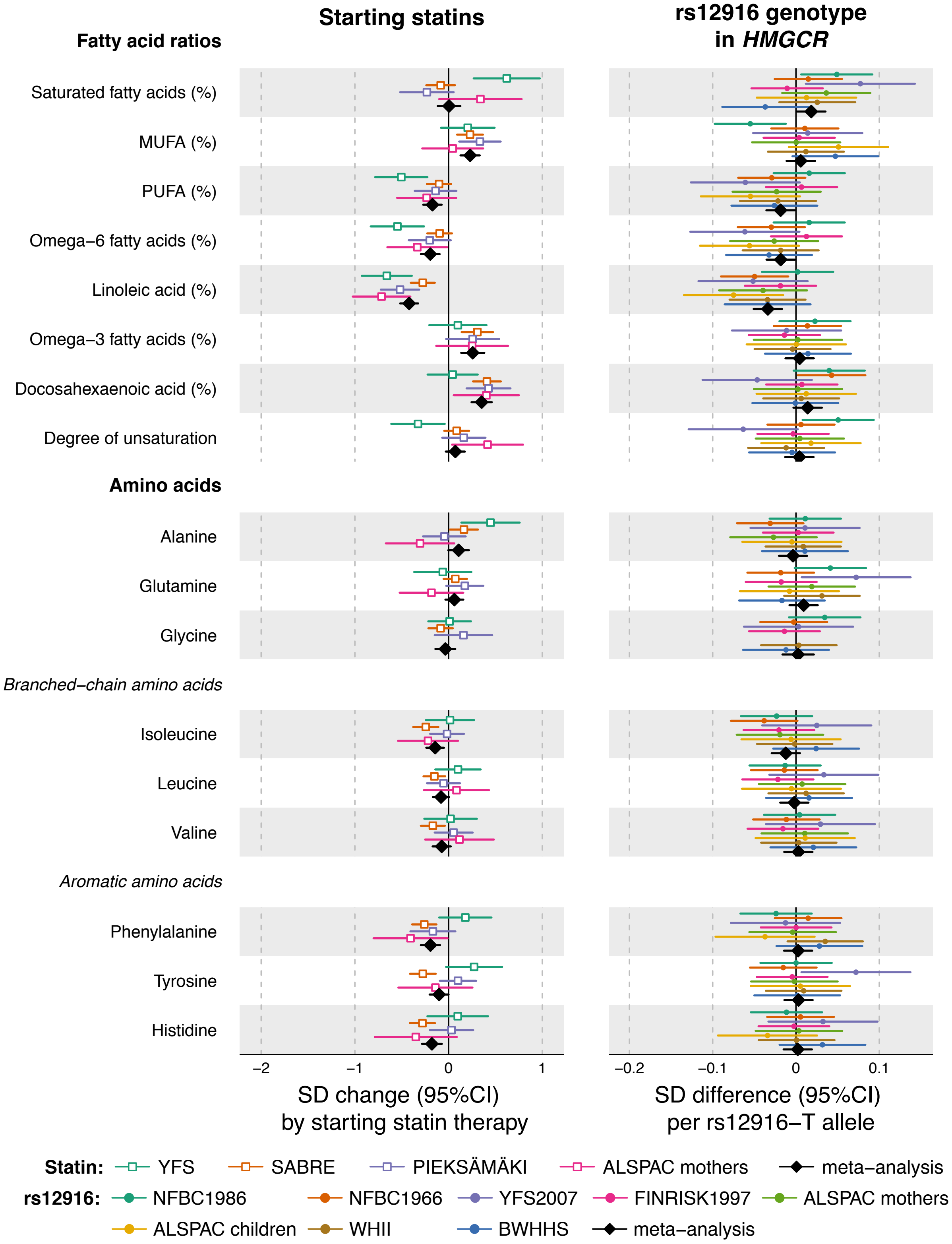

Supplementary Figure 3E. Left: Fatty acid and metabolite changes associated with starting statin therapy in the four cohorts with profiling at two time-points. Associations were adjusted for age and sex. Results are shown in SD-scaled concentration units.

Right: Fatty acid and metabolite associations with rs12196 in *HMGCR* in the eight individual cohorts. Associations were adjusted for age, sex, and population stratification. Results are shown in SD-scaled concentration units.

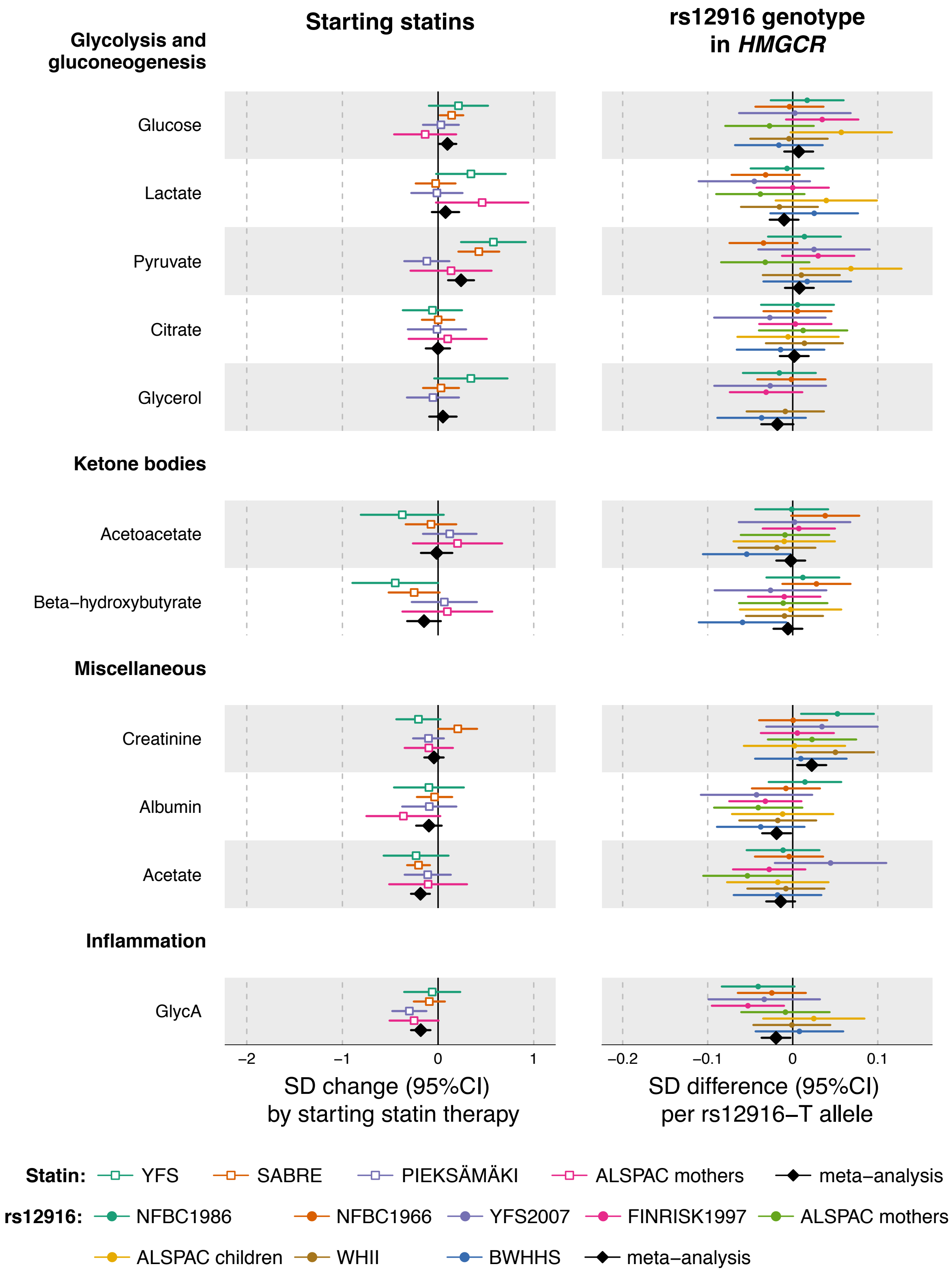

Supplementary Figure 3F. Left: Metabolite changes associated with starting statin therapy in the four cohorts with profiling at two time-points. Associations were adjusted for age and sex. Results are shown in SD-scaled concentration units.

Right: Metabolite associations with rs12196 in *HMGCR* in the eight individual cohorts. Associations were adjusted for age, sex, and population stratification. Results are shown in SD-scaled concentration units.

Supplementary Figure 4. Lipid and metabolite changes associated with starting statin therapy when adjusted for additional cardiovascular risk factors.

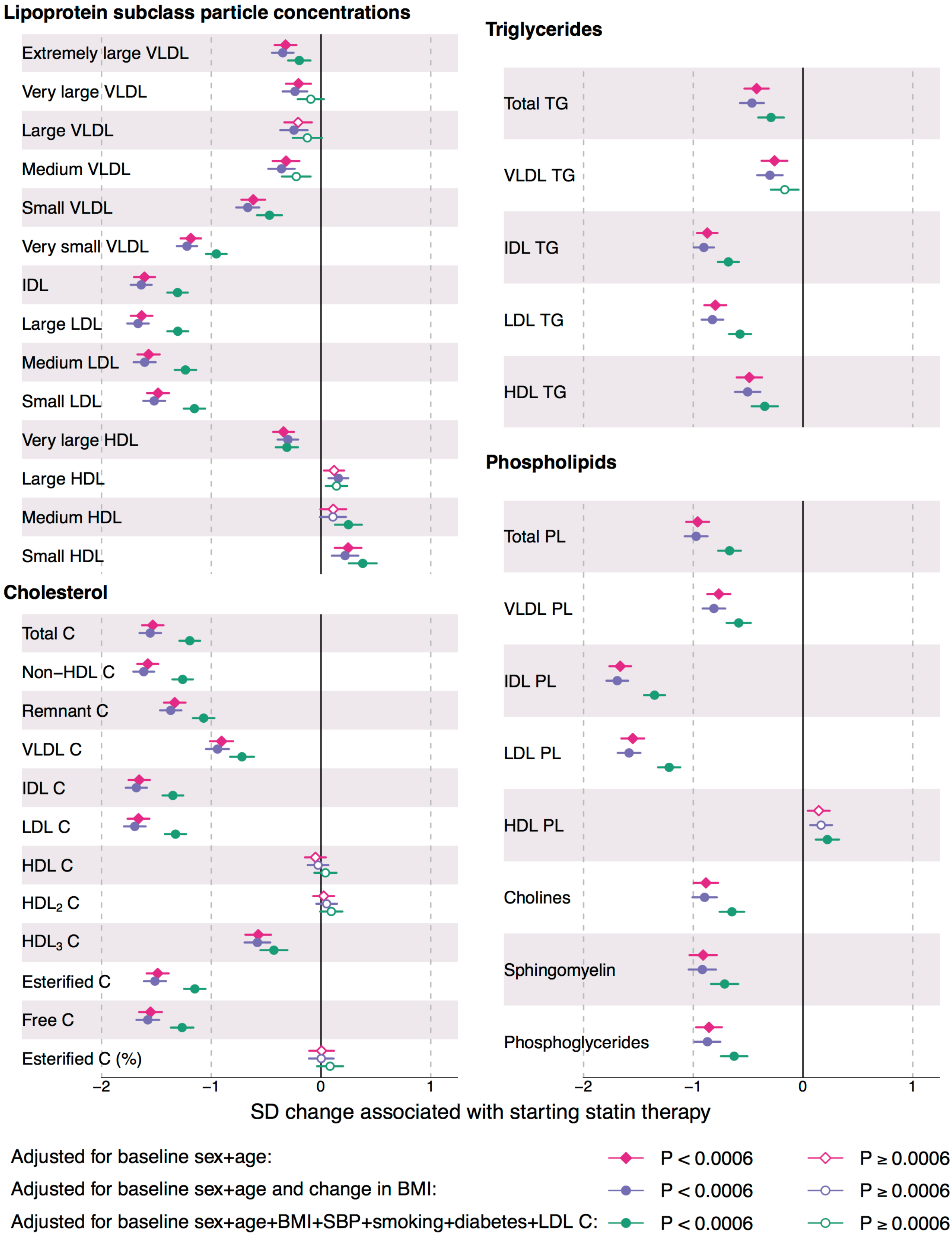

Supplementary Figure 4A: Lipid changes associated with starting statin therapy according to three models: 1) adjusted for age and sex (magenta); 2) adjusted for age, sex and change in BMI during follow-up (purple); and 3) adjusted for age, sex and baseline BMI, systolic blood pressure, smoking status, diabetes status and LDL-C (green). Results were meta-analyzed for the four longitudinal cohorts. Association magnitudes are shown in SD-scaled concentration units. Error bars denote 95% confidence intervals.

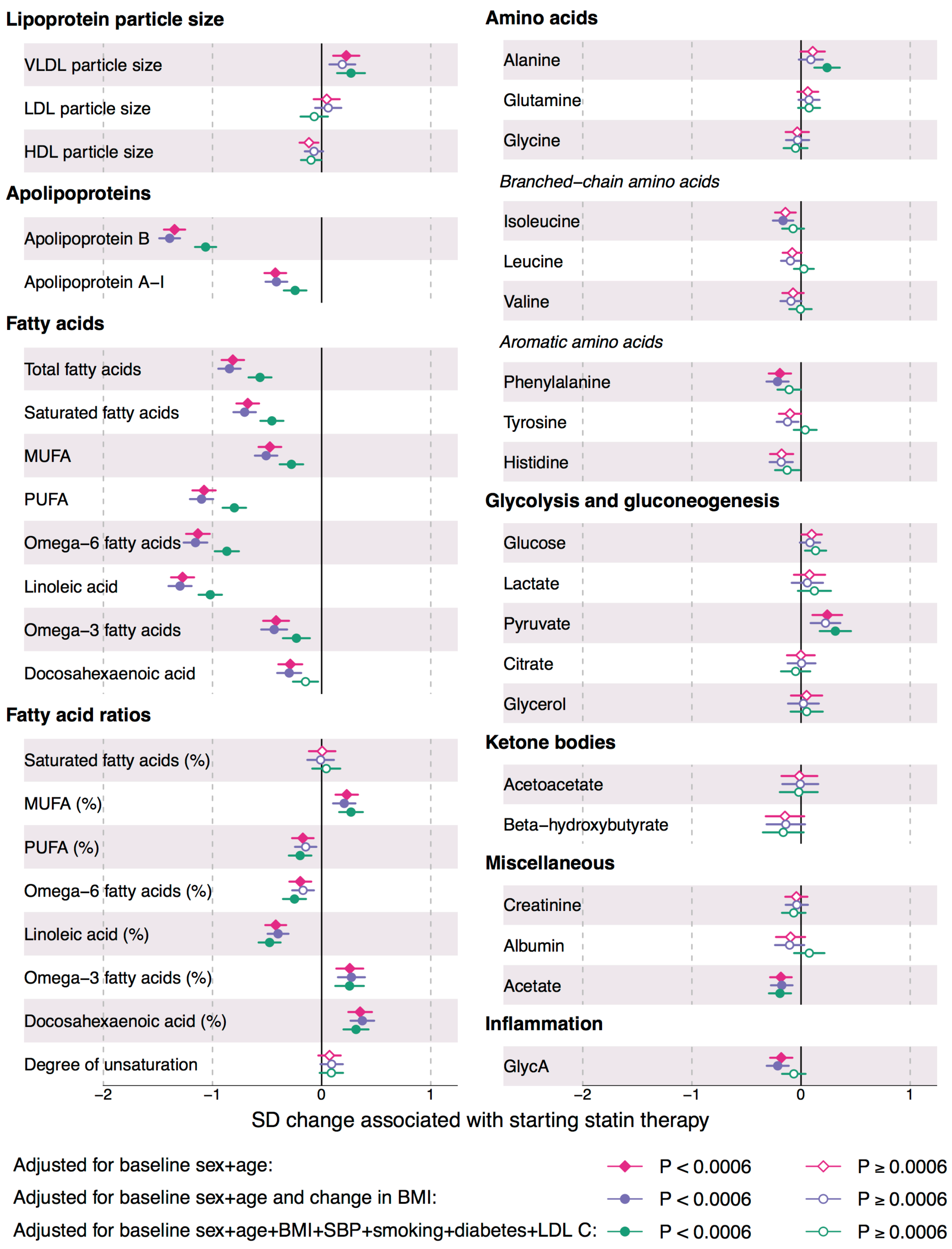

Supplementary Figure 4B: Lipid and metabolite changes associated with starting statin therapy according to three models: 1) adjusted for age and sex (magenta); 2) adjusted for age, sex and change in BMI during follow-up (purple); and 3) adjusted for age, sex and baseline BMI, systolic blood pressure, smoking status, diabetes status and LDL-C (green). Results were meta-analyzed for the four longitudinal cohorts. Association magnitudes are shown in SD-scaled concentration units. Error bars denote 95% confidence intervals.

Supplementary Figure 5. Comparison of lipid and metabolite associations for two common genetic variants in the *HMGCR* gene.

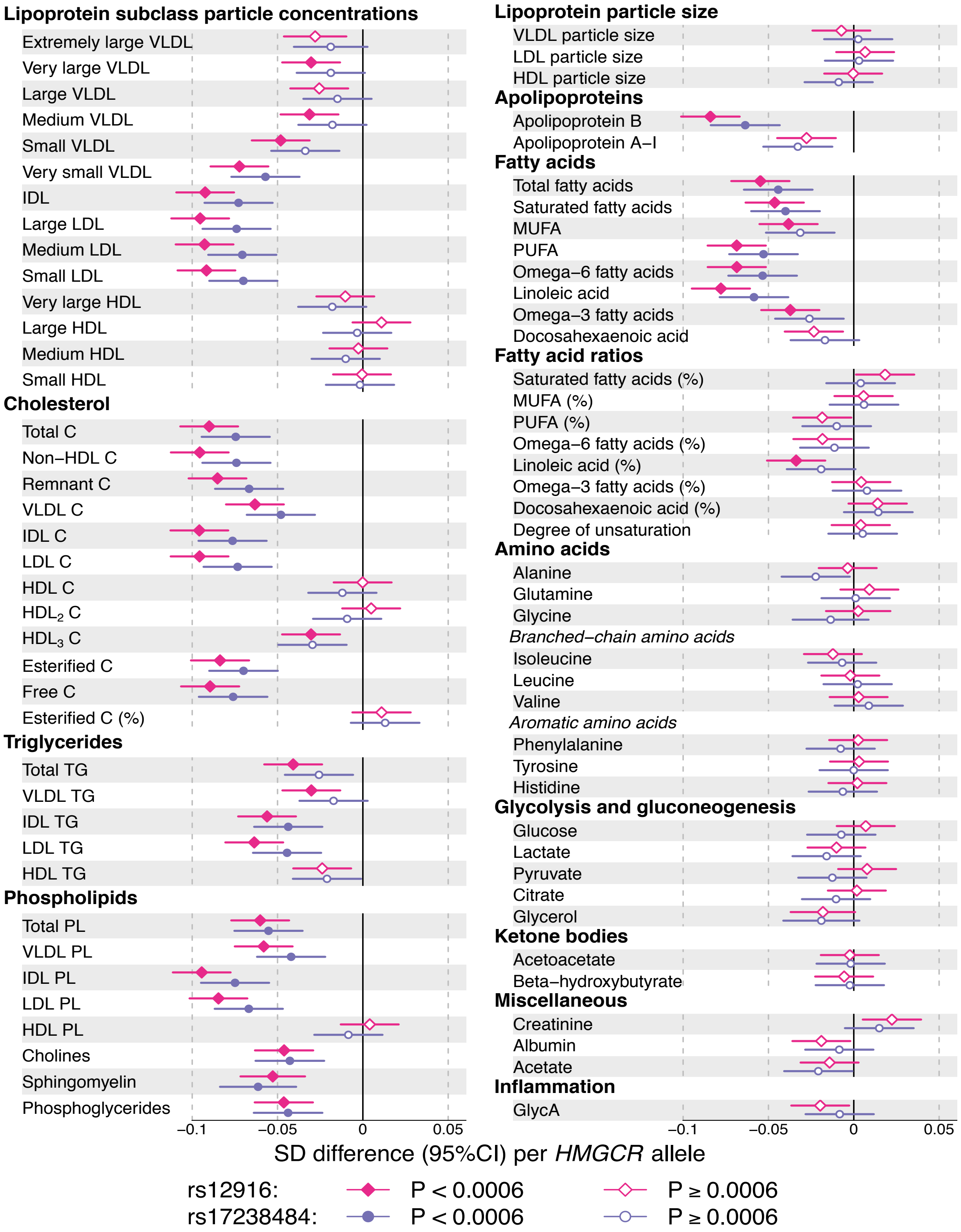

Lipoprotein, fatty acid and metabolite associations with two SNPs, rs12196 and rs17238484, in the *HMGCR* gene (linkage-disequilibrium R^2=0.35). The results are for meta-analysis in the eight population-based cohorts (n=27,914). The metabolic associations were assessed by linear regression models adjusted for age, sex and population stratification. Error bars denote 95% confidence intervals. rs3761742 was used as proxy for rs17238484 for NFBC-1986 and BWHHS (linkage disequilibrium R^2=1). rs698912 was used as proxy for rs17238484 in the ALSPAC children and mothers cohorts (linkage disequilibrium R^2=1).
